# Uncovering the mechanisms of homologous point acupuncture on knee osteoarthritis through an integrated study of metabolomics and proteomics

**DOI:** 10.3389/fbioe.2026.1791109

**Published:** 2026-04-22

**Authors:** Wu Liu, Min Tang, Qi Zhang, Zhaoduan Hu, Rui Peng, Mi Huang

**Affiliations:** 1 College of Acupuncture-Moxibustion and Orthopedics, Hubei University of Chinese Medicine, Wuhan, Hubei, China; 2 Hubei Shizhen Laboratory, Wuhan, Hubei, China; 3 Orthopedics Department, Wuhan Hospital of Traditional Chinese and Western Medicine, Wuhan, Hubei, China

**Keywords:** apoptosis, autophagy, glucose metabolism, homologous point acupuncture, knee osteoarthritis, NLRP3

## Abstract

**Background:**

The incidence and prevalence of knee osteoarthritis (KOA) are on the rise, with the intricate etiology and pathogenesis of the disease posing significant challenges to global health. Although acupuncture has significant promise in modulating the body’s metabolism and inflammatory reactions, the efficacy of various acupuncture methods for treating KOA remains uncertain. In this research, we evaluated the efficacy of homologous point acupuncture compared to conventional acupuncture for KOA and investigated the potential therapeutic mechanisms of homologous point acupuncture through a rat model.

**Methods:**

Rat models of KOA were established by intra-articular injection of monosodium iodoacetate. The successfully modeled rats were allocated into three groups: KOA group (model group, injected with MIA), conventional acupuncture group (AP), and homologous point acupuncture group (HAP). Additionally, healthy rats were designated as a normal control group (Control), with ten rats in each group. Following 4 weeks of consecutive acupuncture treatment, joint swelling and Lequesne MG scores were assessed in each group of rats. Subsequently, blood samples were obtained from the abdominal aorta to assess the expression levels of IL-6, IL-1β, TNF-α, and LDH. Integrated metabolomics and proteomics reveal the mechanism of homologous point acupuncture in KOA rats. Validation of these mechanisms included staining with hematoxylin and eosin, ELISA, biochemical kit, and Western blotting.

**Results:**

Behavioral assessments revealed that, compared to the control group, Lequesne MG scores and degree of joint swelling were significantly increased in the KOA group, whereas both AP and HAP treatment significantly decreased these parameters. Histopathological examination indicates that the cartilage layer in KOA rat models exhibits thinning, irregular surface texture, and indistinct tidemark. In contrast to the KOA group, both AP and HAP exhibited reduced articular cartilage surface irregularity and mitigated cartilage degeneration, and a notable decrease in the levels of IL-6, IL-1β, TNF-α, and LDH in the rat serum following treatment. Further analysis through metabolomics and proteomics reveal that HAP exerts its therapeutic effects by modulating pathways associated with immunity and metabolism, with specific emphasis on Arachidonic acid metabolism and glycolytic processes. Western blot analysis demonstrated that HAP decreased the expression levels of proteins involved in glycolysis, NLRP3 inflammasome activation, and autophagy and apoptosis, and increased the levels of proteins that promote autophagy and prevent apoptosis.

**Conclusion:**

HAP significantly reduced pain-related responses and joint swelling in KOA rats. Specifically, HAP manifests inhibitory effects on inflammatory response in KOA and alleviates knee joint damage in a rat model. These effects are associated with the modulation of glycolysis, NLRP3 inflammasome activation, autophagy, and apoptosis. However, due to the absence of a sham acupuncture control, the specific therapeutic contribution of needling itself cannot be distinguished from non-specific effects.

## Introduction

Osteoarthritis (OA) is a chronic joint disease marked by the degeneration and degradation of articular cartilage, along with synovitis, changes in the composition of synovial fluid, subchondral bone remodeling, and osteophyte formation. It predominantly affects the knee, hip, and spinal joints, with knee osteoarthritis (KOA) being the most prevalent (Hunter and Bierma-Zeinstra, 2019). KOA presents with joint pain that intensifies during movement and eases with rest, along with episodes of transient or self-limiting joint stiffness. Escalated pain and functional limitations can profoundly impair patients’ quality of life (Gelber, 2024). The worldwide prevalence of OA among middle-aged and elderly populations is 22.9%, with an especially sharp increase in KOA prevalence and incidence among individuals over 55 in China, where rates are notably higher in women than in men (Cui et al., 2020; Chen et al., 2021). While KOA primarily results from degenerative alterations in the joints, it is nonetheless linked to various factors including age, body weight, sex, physical activity, and joint trauma (Dong et al., 2023; Zhu S. et al., 2024). Currently, the treatment of KOA includes pharmacological therapies such as non-steroidal anti-inflammatory drugs, opioids, corticosteroids, and hyaluronic acid injections. However, these medications are primarily used to alleviate pain and improve function, and they cannot prevent disease progression. Long-term use may lead to gastrointestinal discomfort, drug resistance, and other adverse events (Primorac et al., 2021; Wei et al., 2023). KOA rehabilitation encompasses exercise therapy, dietary adjustments, biomechanical interventions, and gait training, with personalized treatment plans devised to alleviate pain and enhance limb function; however, there is a lack of robust clinical evidence to substantiate these approaches (Marriott and Birmingham, 2023). In cases of advanced KOA where conservative treatment proves ineffective, surgical options such as joint replacement are advised, though these procedures entail inherent surgical risks and potential postoperative complications (Beswick et al., 2019). Consequently, there are many limitations to the current treatment of KOA, making the exploration of novel therapeutic approaches crucial for advancing its management.

Osteoarthritis (OA) is a whole-joint disorder characterized by progressive degeneration and structural alterations across all articular tissues, including subchondral bone remodeling, meniscal degeneration, inflammatory and fibrotic changes in the infrapatellar fat pad and synovium, as well as metabolic dysregulation and inflammatory responses in chondrocytes (Jiang, 2022; Pereira Herrera et al., 2025; Lian et al., 2019; Ramasamy et al., 2021). During the pathogenesis of KOA, dysregulation of chondrocyte energy metabolism results in oxidative stress and cell apoptosis. Improving cellular metabolic patterns to maintain the homeostasis of chondrocytes contributes to the repair and regeneration of bone tissue (Court et al., 2024; Chen Y. et al., 2024). Chronic or low-grade inflammation in osteoarthritis is primarily mediated by the immune system, with local or systemic inflammation causing cartilage degeneration, bone remodeling, infrapatellar fat pad and synovial changes, thus targeting inflammatory responses can be a therapeutic approach for KOA (De Roover et al., 2023). Glycolysis, a key component of glucose metabolism, exerts a substantial impact on the biological processes of inflammation, apoptosis, and pyroptosis in KOA, making the modulation of various rate-limiting enzymes in glycolysis a novel therapeutic target for KOA treatment (Jiang et al., 2024a). NLRP3 inflammasome-associated proteins trigger KOA inflammatory responses by promoting IL-1β secretion, and suppressing the activation of the NLRP3 inflammasome can reduce damage associated with KOA (Chang et al., 2024). Furthermore, in the pathogenesis of KOA, chondrocyte autophagy and apoptosis are involved throughout the entire process and are regulated by various signaling pathways and Non-Coding RNA (Kao et al., 2022; Wang et al., 2023). Consequently, addressing chondrocyte metabolic imbalances, curbing inflammatory reactions, and modulating autophagy and apoptosis to enhance chondrocyte proliferation could be a new therapeutic approach for KOA.

Acupuncture offers distinctive benefits for analgesia, and researchers have used molecular biology, histopathology, and imaging techniques to clarify the mechanisms of acupuncture analgesia (Wang et al., 2022; Wu et al., 2024). For instance, electroacupuncture can suppress the release of downstream inflammatory mediators and matrix metalloproteinases via the TLRs/NF-κB signaling pathway and also alleviate KOA injury by inhibiting NLRP3 inflammasome-induced pyroptosis (Ruan et al., 2021; Zhang et al., 2023). Fire needle enhances the recovery of synovial and chondral tissue injury through modulating macrophage polarization (Wei et al., 2022). Furthermore, acupuncture reduces joint pain through the regulation of resting-state functional connectivity of the ventrolateral periaqueductal gray (vlPAG) in the brain (Zhou et al., 2023). Consequently, further research into the mechanisms of acupuncture treatment for KOA can provide more evidence for acupuncture analgesia.

Homologous point acupuncture (HAP) is an acupuncture technique guided by the traditional Chinese medicine theory of “Yangyuan Tongluo”, commonly used for the treatment of pain-related diseases (Xie et al., 2022). The HAP therapy, proposed by Professor Peng Rui, is grounded in visceral-meridian theory, it innovatively incorporates Jiaji points at dorsal segmental levels homologous to Back-Shu points, alongside traditional acupoints (Peng, 2022). Through multidimensional syndrome differentiation, Jiaji points undergo swift needle penetration without retention, while acupoints along limb meridians receive conventional needling without requiring tonification or reduction techniques. In clinical practice, we have achieved satisfactory therapeutic effects using HAP for the treatment of KOA. Metabolic dysregulation in chondrocytes and inflammatory reactions are primary pathological elements in the onset of KOA, involving processes of autophagy and apoptosis. However, it remains unclear whether HAP affects the aforementioned pathological factors in KOA.

This research investigated the molecular mechanisms underlying the intervention of HAP in a KOA rat model. By employing histopathological, molecular biological, metabolomic, and proteomic techniques, the study established the mechanisms of action of HAP in the KOA model and determined its regulatory effects on immune metabolism. The objective of this research is to assess the efficacy of HAP in a KOA rat model, clarify the possible mechanisms underlying its intervention, and establish the evidence base for its use in KOA treatment.

## Materials and methods

### Experimental animals and grouping

All male Sprague-Dawley (SD) rats, aged 8–10 weeks and weighing 200 ± 20 g, were sourced from Wuhan Purui Zhiyi Biotechnology Co., Ltd., and maintained at the Experimental Animal Center of Hubei Provincial Disease Prevention and Control Center. The breeding room was kept at a temperature of 22 °C–25 °C, humidity was controlled at 55% ± 5%, and there was a 12-h cycle of light and darkness from fluorescent lighting. The animals had *ad libitum* access to standard pelleted diet and water. After a 7-day acclimation period, rats were randomly divided into the healthy control group (Control), KOA group (KOA), conventional acupuncture group (AP), and HAP group (HAP), with 10 rats per group. Sample sizes were determined based on previous experience with the MIA-induced KOA model and feasibility considerations. For omics analyses (metabolomics and proteomics), six biological replicates per group were used, which is consistent with exploratory multi-omics studies in rodent models. However, no formal power calculation was performed prior to the experiment; therefore, the omics findings should be considered exploratory and hypothesis-generating rather than confirmatory. Following the grouping, Roman numerals were marked on the tails of the rats for identification. The animal experiment conducted herein has received approval from the Hubei Provincial Disease Prevention and Control Center’s Committee on Laboratory Animal Management and Use (Approval No.: 202420281).

### Preparation of KOA model rats

Animals were anesthetized using isoflurane at an induction concentration of 4% (Batch number: Shou Yao Zi 153717015; provided by Qingdao Oubofang Medical Technology Co., Ltd.), and the anesthesia was maintained at a concentration of 2%–3% isoflurane. Post-induction, the rats were affixed to the surgical board. The monosodium iodoacetate (MIA) was employed to establish the KOA model in rats. Using a microinjector, 50 μL of 0.9% physiological saline containing 3 mg of MIA was injected into the intra-articular cavity of the right knee. Following the manipulation of joint flexion and extension, the rats were placed back into their breeding cages (Jiang X. et al., 2024). Following a week of regular feeding post drug administration, signs such as swelling of the right knee joint, restricted joint movement, and limping in the model rats suggested a successful model creation.

### Acupuncture intervention

Following anesthesia with isoflurane (2%–4% concentration) in AP group, rats were subjected to needling at the right posterior knee’s Dubi (ST35), Neixiyan (EX-LE4), Yanglingquan (GB34), and Zusanli (ST36) points. The needles used were 0.18*13 mm in size (Batch number: 20162270970; Suzhou Medical Supplies Factory Co., Ltd.), inserted into the acupoints to a depth of 3–4 mm. Acupoints were selected based on the methods described in Experimental Acupuncture (Guo, 2021). Following anesthesia, the rats in HAP group, apart from the previously mentioned acupoints, were selected for the segmental Jiaji points associated with the liver Shu (BL18), gallbladder Shu (BL19), spleen Shu (BL20), and stomach Shu (BL21), positioned beside the intercostal spaces under the 9th to 12th thoracic vertebrae, 0.3 cm from the paramedian line on the dorsal side. Acupuncture operations for ST35, EX-LE4, GB34, and ST36 are consistent with those of the AP group, complemented by quick puncture of the Jiaji points without leaving the needles in, with a depth of 3–4 mm. Needle acupuncture commenced 1 week post-modeling, administered once daily, with 1 week constituting one treatment course, and the intervention lasted for a total of 4 weeks.

### Sample collection

**FIGURE 1:**
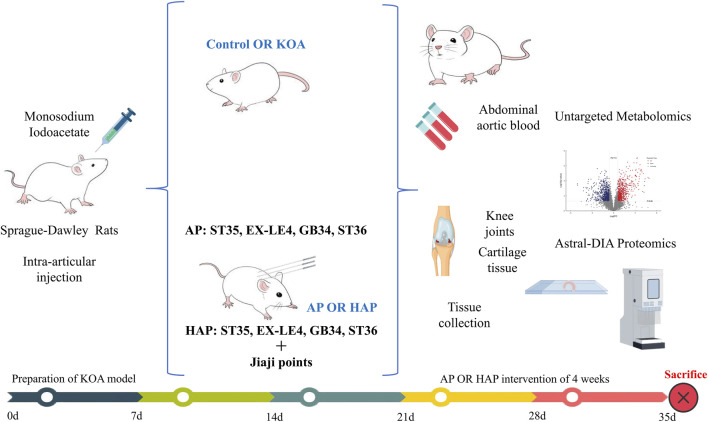
The flow chart of the animal experiment. Abbreviations: AP, conventional acupuncture; HAP, homologous point acupuncture.

Following the last intervention, 24 h post-treatment, 5 mL of rat abdominal aortic blood was aseptically collected, centrifuged at 3000 rpm for 10 min to obtain the serum for subsequent measurements. The rats were subsequently euthanized via dislocation, and the cartilage tissue beneath the patella in the right knee joint was extracted and preserved at −80 °C for subsequent analysis. From some rats, the entire right knee joints (1 cm above and below the joint) were collected and treated with a 10% EDTA solution to decalcify for 4 weeks. Once the bone tissue had softened, the samples were dehydrated, embedded in paraffin, and then stained with HE (Figure 1).

### Lequesne MG scores and degree of joint swelling

The Lequesne MG scores was assessed after intervention. This scoring system evaluates three parameters: pain or discomfort response, degree of daily activity limitation, and joint swelling. The total score ranges from 0 to 12 points, with higher scores indicating more severe arthritis symptoms. Knee joint diameter was measured using a standard ruler before modeling and after intervention. The measurement was taken at the level of the white ligament of the knee joint. Joint swelling (%) was calculated as: [(knee diameter after intervention - knee diameter before modeling)/knee diameter before modeling] × 100%.

### Hematoxylin and eosin (H&E), Mankin score and Krenn score

Decalcified mouse knees were fixed in 4% paraformaldehyde, followed by dehydration before embedding in paraffin to make 5‐µm sections. Sections were deparaffinized with xylene, dehydrated with gradient ethanol and then stained with H&E. Histopathological evaluation of H&E-stained sections included Mankin score and Krenn synovitis score. The Mankin score (scale: 0–14) evaluates cartilage structure, chondrocytes, matrix staining, and tidemark integrity, with higher scores indicating more severe degeneration. The Krenn score (scale: 0–9) quantifies synovial inflammation based on synovial lining cell layer, stromal cell density, and inflammatory infiltrate.

### Enzyme linked immunosorbent assay (ELISA)

ELISA was utilized to quantify concentrations of pro‐inflammatory factors IL‐1β (MM-0047R2, WUHAN HUAYAN Biotechnology CO., LTD), IL‐6(MM-0190R2, WUHAN HUAYAN Biotechnology CO., LTD), and TNF‐α (MM-0180R2, WUHAN HUAYAN Biotechnology CO., LTD) in the serum, using commercial kits according to the manufacturer’s instructions. Instrumentation included: Microplate reader (Multiskan™ FC, Thermo Fisher Scientific), High-speed refrigerated centrifuge (H1-16KR, Hunan Xiangyi), Thermostatic incubator (AS ONE Corp., Japan), Automated microplate washer (AMW-3000, LabTech), Multi-sample tissue homogenizer (Tissuelyser-24L, Shanghai Jingxin).

### Biochemical tests

Lactate dehydrogenase (LDH) biochemical kit (A020-two to two, Nanjing Jiancheng Bioengineering Institute) was used to measure the activity of LDH in serum.

### Untargeted metabolomics

The serum samples were thawed at 4 °C and 100 μL aliquots were mixed with 400 μL of cold methanol/acetonitrile (1:1, v/v) to remove the protein. The mixture was centrifuged for 20 min (14000 rpm, 4 °C). The supernatant was dried in a vacuum centrifuge. For LC-MS analysis, the samples were re-dissolved in 100 μL acetonitrile/water (1:1, v/v) solvent and centrifuged at 14000 rpm at 4 °C for 15 min, then the supernatant was injected.

LC-MS/MS Analysis was performed using an UHPLC (1290 Infinity LC, Agilent Technologies) coupled to a quadrupole time-of-flight (AB Sciex TripleTOF 6600). For HILIC separation, samples were analyzed using a 2.1 mm × 100 mm ACQUIY UPLC BEH Amide 1.7 µm column (waters, Ireland). In both ESI positive and negative modes, the mobile phase contained A = 25 mM ammonium acetate and 25 mM ammonium hydroxide in water and B = acetonitrile. The gradient was 95% B for 0.5 min and was linearly reduced to 65% in 6.5 min, and then was reduced to 40% in 1 min and kept for 1 min, and then increased to 95% in 0.1 min, with a 3 min re-equilibration period employed.

The ESI source conditions were set as follows: Ion Source Gas1 (Gas1) as 60, Ion Source Gas2 (Gas2) as 60, curtain gas (CUR) as 30, source temperature: 600 °C, IonSpray Voltage Floating (ISVF) ± 5500 V. In MS only acquisition, the instrument was set to acquire over the m/z range 60–1000 Da, and the accumulation time for TOF MS scan was set at 0.20 s/spectra. In auto MS/MS acquisition, the instrument was set to acquire over the m/z range 25–1000 Da, and the accumulation time for product ion scan was set at 0.05 s/spectra. The product ion scan is acquired using information dependent acquisition (IDA) with high sensitivity mode selected. The parameters were set as follows: the collision energy (CE) was fixed at 35 V with ±15 eV; declustering potential (DP), 60 V (+) and −60 V (−); exclude isotopes within 4 Da, candidate ions to monitor per cycle: 10.

### Astral-DIA quantitative proteomics

An appropriate quantity of cartilage tissue samples were first homogenized by MP FastPrep-24 homogenizer (24 × 2, 6.0M/S, 60 s, twice), and then SDT buffer (4% SDS, 100 mM Tris-HCl, pH 7.6) was added. The lysates were further sonicated, and boiled for 15 min. After centrifuged at 14000 rpm for 40 min, the supernatant was quantified with the BCA Protein Assay Kit. 15 μg of protein for each sample were mixed with 5X loading buffer respectively and boiled for 5 min. The proteins were separated on 4%–20% SDS-PAGE gel (constant voltage 180 V, 45 min). Protein bands were visualized by Coomassie Blue R-250 staining. An equal aliquot from each sample in this experiment was pooled into one sample for quality control.

The samples were subjected to trypsin digestion using the Filter-Aided Sample Preparation (FASP) technique (Wiśniewski et al., 2009). The peptides of each sample were desalted on C18 Cartridges (Empore™ SPE Cartridges MCX, 30UM,waters), concentrated by vacuum centrifugation and reconstituted in 40 µL of 0.1% (v/v) formic acid. The peptide content was estimated by UV light spectral density at 280 nm. For DIA experiments, iRT calibration peptides were spiked into the sample. Mass Spectrometry Assay for Data Independent Acquisition (DIA): The peptides from each sample were analyzed by OrbitrapTM AstralTM mass spectrometer (Thermo Scientific) connected to an Vanquish Neo system liquid chromatography (Thermo Scientific) in the data independent acquisition (DIA) mode. Precursor Ions were scanned at a mass range of 380–980 m/z, MS1 resolution was 240000 at 200 m/z, Normalized AGC Target: 500%, Maximum IT: 5 ms. 299 windows were set for DIA mode in MS2 scanning, Isolation Window: 2m/z,HCD Collision Energy: 25ev, Normalized AGC Target: 500%, Maximum IT: 3 ms.

### Western blotting

Proteins were extracted from the collected cartilage tissue samples using pre‐cooled RIPA lysis buffer supplemented with a protease/phosphatase inhibitor cocktail and PMSF (MA0151, Meilunbio). Protein quantification was performed using the BCA assay. Sodium dodecyl sulfate polyacrylamide gel electrophoresis (SDS-PAGE) was utilized for protein separation, followed by transfer to a polyvinylidene fluoride (PVDF) membrane (IPVH00010, Millipore). Blocked with 5% bovine serum albumin, and incubated with diluted primary antibody overnight, including GAPDH (dilution concentration:1:10000), HIF1α (dilution concentration:1:1000), HK2 (dilution concentration: 1:1000), c-caspase3 (dilution concentration: 1:1000), Bax (dilution concentration: 1:10000), LC3 (dilution concentration: 1:1000), Beclin1 (dilution concentration: 1:6000), NLRP3 (dilution concentration: 1:1000), ASC (dilution concentration: 1:1000), caspase1 (dilution concentration: 1:1000), p62 (dilution concentration: 1:5000), Bcl2 (dilution concentration: 1:5000). (Note: GAPDH was used as the sole housekeeping protein for normalization in this study. While GAPDH is commonly used, its expression can be influenced by metabolic states. Future studies should consider using multiple housekeeping proteins or total protein staining for more robust normalization in metabolic research. Uncropped and unprocessed Western blot images for all figures are provided in Supplementary Figure S1 to ensure data transparency).

All the primary antibodies were validated by either the manufacturer or other available publications, listed as follows: HIF1α (Boster, A00013-1), HK2 (Abclonal, A0994), c-caspase3 (Affinity, AF7022), Bax (Wuhan Sanying, 50599-2-Ig), LC3 (CST, 12741T), Beclin1 (Wuhan Sanying, 11306-1-AP), and GAPDH (Wuhan Sanying, 60004-1-Ig) from WUHAN HUAYAN Biotechnology CO., LTD. NLRP3 (Affinity, BF8029), ASC (Affinity, DF6304), caspase1 (Affinity, AF5418), p62 (Wuhan Sanying, 18420-1-AP), Bcl2 (Wuhan Sanying, 60178-1-Ig) from WUHAN Fabre Biotechnology CO., LTD. After washing 5 times for 5 min each with TBST (T-Pro), and a horseradish peroxidase (HRP)-conjugated secondary antibody (Boster, BA1051 and BA1054) was applied for 2 h at room temperature. Visualized using a commercial enhanced chemiluminescence kit (Affinity, KF8003), Grey values of the obtained bands were quantified using Image-Pro Plus software.

### Statistical analysis

The data were demonstrated as mean ± standard error of the mean (SEM) and analyzed with GraphPad Prism software (version 8.0.1.244). Normality was assessed using the Shapiro-Wilk test prior to parametric testing. For behavioral and biochemical data, comparisons among multiple groups were performed using one-way ANOVA followed by Tukey’s *post hoc* test, with *p* < 0.05 considered statistically significant. For omics data (metabolomics and proteomics), differential metabolites and proteins were identified using criteria of VIP > 1 (from OPLS-DA) and *p* < 0.05, with p-values adjusted for multiple testing using the Benjamini–Hochberg false discovery rate (FDR) method where applicable.

## Results

### HAP reduces the lequesne MG score and joint swelling degree

**FIGURE 2:**
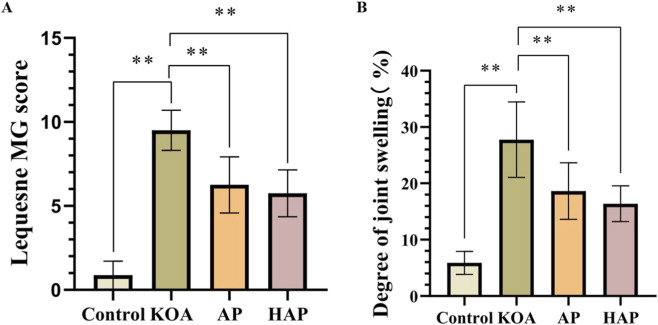
Analysis of effect of HAP against KOA based on behavioral assessment and joint swelling degree. Note: **(A)** Lequesne MG score, **(B)** Degree of Joint swelling. Data are presented as mean ± SEM (n = 8). **p < 0.01.

After the 4-week acupuncture intervention, pain or discomfort response, degree of daily activity limitation, and joint swelling were assessed and recorded in each group. The results showed that, compared to the control group, the KOA group exhibited significantly higher Lequesne MG scores and joint swelling (*p* < 0.01). In contrast, both the AP and HAP groups showed significantly reduced Lequesne MG scores and joint swelling (*p* < 0.01), with no significant difference observed between the two treatment groups (Figure 2).

### HAP possesses anti-inflammatory effects in a rats model of KOA

**FIGURE 3:**
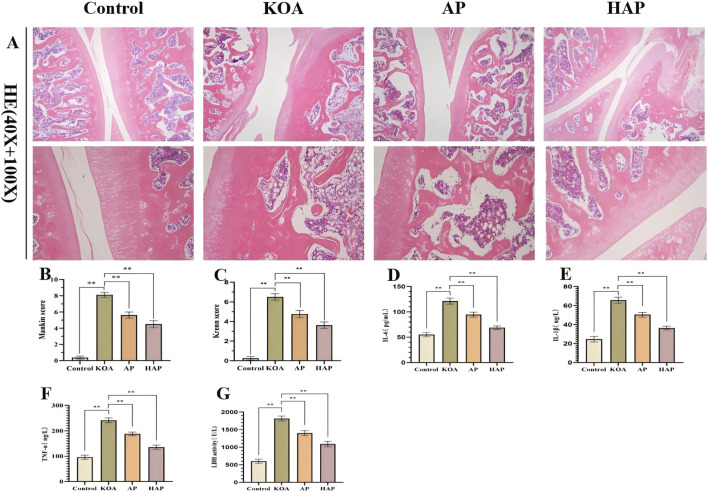
The protective effect of HAP on KOA model rats were evaluated. Note: **(A)** The pathological characteristics of the knee joints were observed by H&E, **(B,C)** Mankin and Krenn Score. **(D–G)** Expression levels of IL-6, IL-1β, TNF-α, and LDH activity in serum. Data are presented as mean ± SEM (n = 8). **p < 0.01, *p < 0.05.

Following a 4-week intervention period, pathological assessments of the knee joints from each group of rats were performed using hematoxylin and eosin staining. In contrast to the control group, the KOA group rats exhibited an irregular surface of the articular cartilage and degenerative changes, demonstrating that MIA induced cartilage degeneration and structural injury. In AP group and HAP group, the irregularity of the joint cartilage surface in rats was decreased, and the degeneration of cartilage was alleviated (Figure 3A). According to the results of the Mankin and scores, OA group was significantly different from control group after treatment (*p* < 0.001). In addition, OA group was apparently different from treatment group (*p* < 0.001) (Figure 3B). OARSI score came to the same results as those of Mankin score (*p* < 0.001) (Figure 3C), suggesting that acupuncture treatment significantly improves the structure and function of knee cartilages and effectively delays the further progression of KOA.

Inflammatory factors and LDH activity are important in the pathology of KOA, therefore, employing ELISA to measure the levels of IL-6, IL-1β, TNF-α, and LDH activity in serum is of significant importance. The findings showed that the KOA group had a significant increase in the concentrations of IL-6, IL-1β, and TNF-α, leading to an intensified inflammatory reaction, accompanied by an increase in LDH activity. Following treatment with conventional AP or HAP, there was a notable decrease in MIA-induced inflammatory cytokines and LDH activity (Figures 3D–G), while HAP showed numerically lower mean values for these parameters, direct statistical comparison between AP and HAP groups was not the primary focus of this study, and the observed trends do not imply statistically significant superiority. A comprehensive multi-omics comparative analysis between AP and HAP is warranted to elucidate potential differences in their underlying mechanisms.

### HAP treatment is associated with altered metabolic profiles in rats with KOA

**FIGURE 4:**
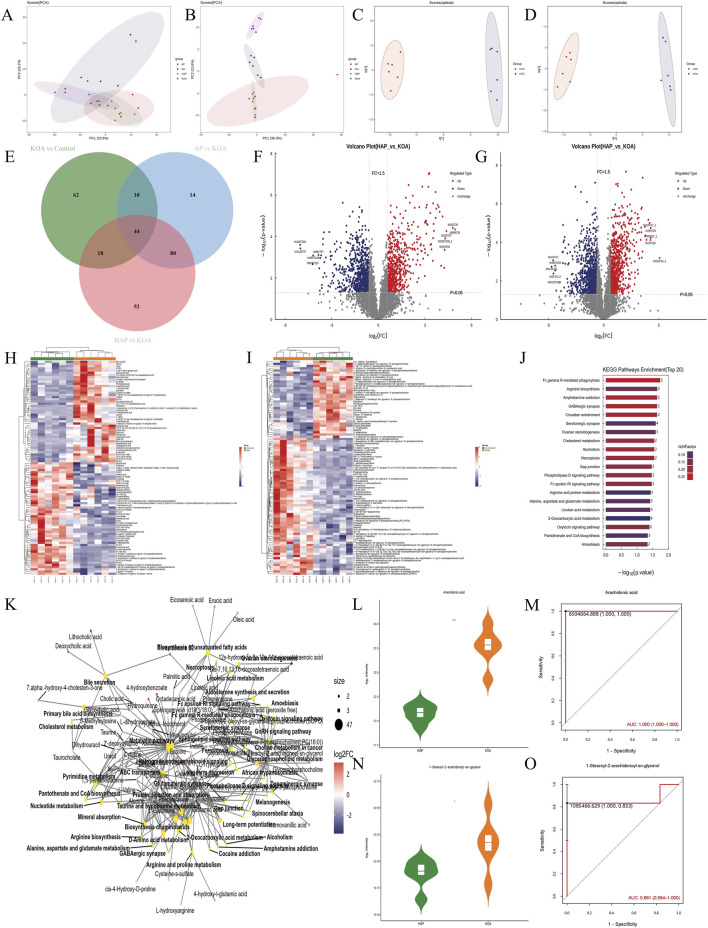
Analysis of target and pathway of HAP against KOA based on metabolomics. Note: **(A,B)** Principal Component Analysis (PCA) plots for each group in positive and negative ion modes. **(C,D)** Orthogonal Partial Least Squares-Discriminant Analysis (OPLS-DA) plots for HAP versus KOA in positive and negative ion modes. **(E)** Venn diagram of differential metabolites among KOA, AP, and HAP groups. **(F,G)** Volcano plots of differential metabolites between HAP and KOA groups under positive and negative ion modes, where red, blue, and gray represent upregulated, downregulated, and unchanged metabolites, respectively. **(H,I)** Hierarchical cluster analysis of differential metabolites between HAP and KOA groups under positive and negative ion modes. **(J)** KEGG enrichment results of differential metabolites between HAP and KOA groups. **(K)** Network diagram of differential metabolic pathways between HAP and KOA, where yellow dots represent pathways, and other dots represent metabolites. The size of the pathway nodes indicates the number of connected metabolites, with more connections leading to larger nodes. The metabolite nodes are represented by a gradient color to denote the magnitude of the log2(FC) values. **(L,N)** Quantitative results of metabolites. **(M,O)** ROC results of metabolites. Compared with KOA, ** and * represent *p* < 0.01 and 0.05, respectively.

In order to gain a deeper understanding of the alterations in metabolic pathways related to the treatment of KOA with HAP, the untargeted metabolomics were employed to analyze serum metabolites, with six biological replicates for each group. Principal Component Analysis (PCA) coupled with Orthogonal Partial Least Squares Discriminant Analysis (OPLS-DA) revealed distinct differences in the metabolic profiles among the Control, KOA, and HAP groups (Figures 4A–D). Differential metabolites were screened based on the criteria of OPLS-DA VIP > 1 and *p* < 0.05. In comparisons of KOA vs. Control, AP vs. KOA, and HAP vs. KOA, 134, 148, and 194 distinct differential metabolites were identified, respectively (Figure 4E; Supplementary Tables 1–3). It is noteworthy that 86 metabolites were significantly increased in the HAP vs. KOA group, comprising Daidzein, 4-pyridoxic acid, L-Serine, L-Glutamine, L-Tryptophan, and other differential metabolites. A total of 108 significantly downregulated metabolites were identified, including Arachidonic acid, 1-Stearoyl-2-arachidonoyl-sn-glycerol, Glycodeoxycholic acid, Lithocholic acid, and Taurine, among other differential metabolites (Figures 4F–I). Through KEGG enrichment analysis, pathways such as Fc gamma R-mediated phagocytosis, Arginine biosynthesis, Fc epsilon RI signaling pathway, Arginine and proline metabolism, and Pantothenate and CoA biosynthesis were significantly enriched, which are related to the immune system and metabolism (Figures 4J,K). Focusing on the immune-related metabolic pathway Fc gamma R-mediated phagocytosis, two metabolites showed significant changes after HAP intervention: Arachidonic acid (AA, AUC = 1) and 1-Stearoyl-2-arachidonoyl-sn-glycerol (1-SAG, AUC = 0.861) were significantly decreased (*p* < 0.01) (Figures 4L–O). Metabolomic results suggest that HAP may exert anti-inflammatory effects by regulating various metabolic pathways, particularly those related to Fc gamma R-mediated phagocytosis, thereby improving the damage in KOA.

### HAP treatment is associated with altered expression of proteins related to immunity and metabolism

**FIGURE 5:**
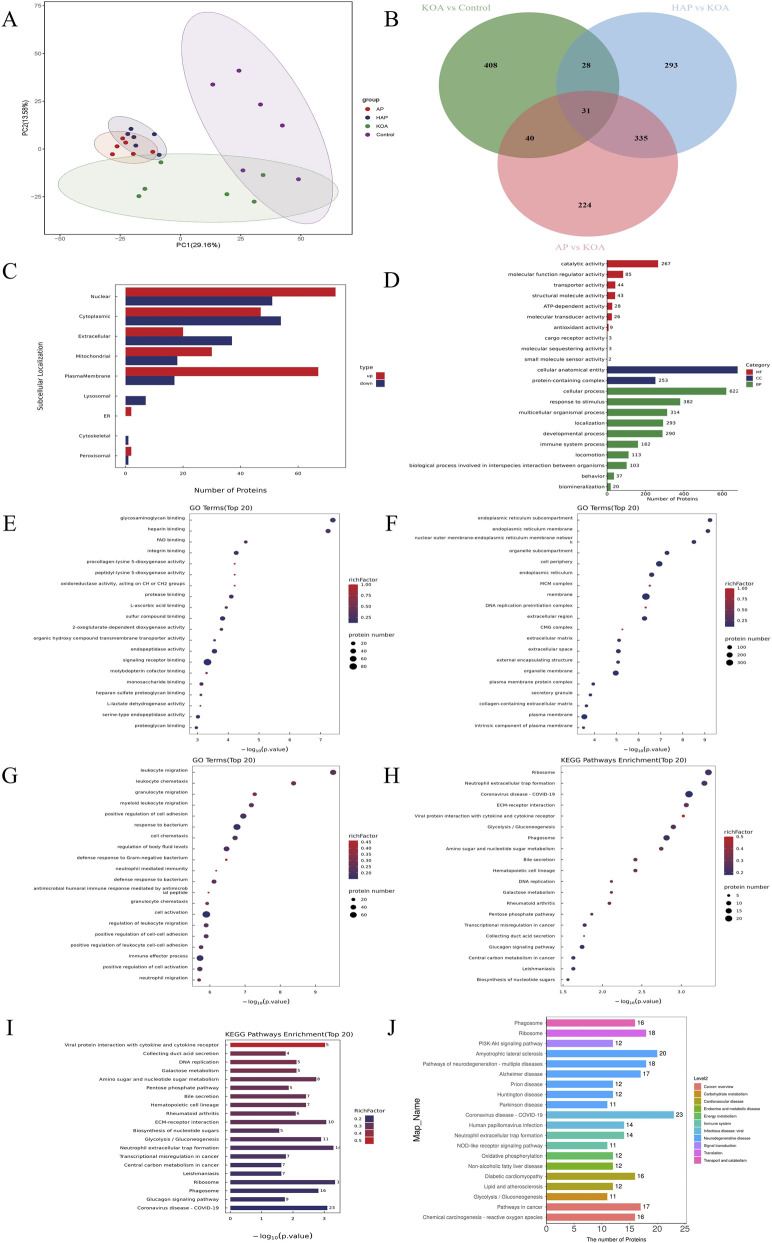
Analysis of target and pathway of HAP against KOA based on astral-based DIA proteomics. Note: **(A)** Principal component analysis (PCA) plots of each group. **(B)** Venn diagram of DEPs between KOA vs. control, AP vs. KOA, and HAP vs. KOA. **(C)** Subcellular structural localization analysis of HAP vs. KOA. **(D)** GO functional analysis of HAP vs. KOA. **(E–G)** GO terms name of molecular function, cellular component, and biological process in HAP vs. KOA. **(H–J)** The KEGG pathway enrichment analysis in HAP vs. KOA.

To further explore the molecular mechanisms of HAP intervention in KOA, Astral-DIA proteomic analysis of cartilage tissue was conducted, with 6 biological replicates per group. Principal component analysis (PCA) revealed distinct protein profiles between AP, HAP, and Control, KOA groups (Figure 5A). To analyze the differences in protein composition among various groups, differentially expressed proteins (DEPs) were screened based on the criteria of Fold Change (FC) > 1.5 (upregulated more than 1.5 times or downregulated less than 0.67 times) and *p* < 0.05. In comparisons of KOA vs. Control, AP vs. KOA, and HAP vs. KOA, 507, 630, and 687 DEPs were identified, respectively. In HAP vs. KOA, this included 346 upregulated proteins and 341 downregulated proteins (Figure 5B; Supplementary Tables 4–6). There were 29 common positive proteins when KOA upregulated and HAP downregulated (*p* < 0.05 and fold-change >1.5), while 14 common positive proteins were found which KOA downregulated and HAP-upregulated (*p* < 0.05 and fold-change >1.5) (Supplementary Table 7). HAP vs. KOA differential proteins were found to mainly belong to nuclear, cytoplasmic, extracellular, mitochondrial, and plasma membrane by subcellular localization (Figure 5C). Based on GO functional analysis, HAP vs. KOA differential proteins were mainly binding, catalytic activity, and molecular function regulator activity proteins related to molecular function (MF), cellular anatomical entity and protein-containing complex proteins related to cellular component (CC), and cellular process, metabolic process, and immune system process proteins related to biological process (BP) (Figures 5D–G). The DEPs were annotated by KEGG pathways, and these DEPs in HAP vs. KOA participated in 37 pathways. The top 20 pathways between pairs of groups (HAP vs. KOA) were plotted (Figures 5H–J). The high representation of neutrophil extracellular trap formation, hematopoietic cell lineage, rheumatoid arthritis pathways suggests changes in the immune system in HAP rats. Enrichment in glycolysis, amino sugar and nucleotide sugar metabolism, galactose metabolism, and oxidative phosphorylation pathways points to effects of HAP on carbohydrate metabolism and energy metabolism in rats.

### HAP suppresses the expression of proteins related to glycolysis and the NLRP3 inflammasome

**FIGURE 6:**
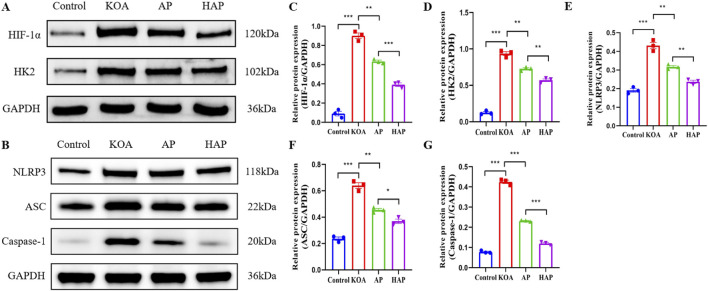
Effects of HAP on cartilage metabolism and inflammation in KOA model rats. Note: **(A,C,D)** Expression of HIF-1α and HK2 in cartilage tissue (GAPDH as internal control). **(B,E–G)** Expression of NLRP3, ASC and Caspase-1 in cartilage (GAPDH as internal control). ***p < 0.001, **p < 0.01, *p < 0.05.

Glycolysis is closely related to metabolic disorders in OA cartilage. In the pathological process of OA, the glycolytic process of chondrocytes is accelerated, leading to the upregulation of various inflammatory factors (Adam et al., 2024). Hypoxia-inducible factor-1α (HIF-1α) and hexokinase 2 (HK2) are crucial in the glycolytic pathway. HIF-1α is the primary transcription factor that promotes glycolysis and can increase the expression of key glycolytic enzymes such as HK2, leading to enhanced apoptosis of chondrocytes and exacerbated inflammatory responses in OA (Liu et al., 2024; Jiang et al., 2024b). In addition, the NLRP3 inflammasome is intimately linked to inflammatory responses, and suppression of NLRP3 inflammasome activation can ameliorate OA cartilage degeneration and inflammatory responses (Xiao et al., 2023). Notably, the suppression of glycolysis can attenuate the activation of the NLRP3 inflammasome (Stanton et al., 2024). Consequently, treatment strategies aimed at HIF-1α, HK2, and proteins associated with the NLRP3 inflammasome could represent a significant direction for the management of KOA. Western blot analysis in this study demonstrated that the protein expression levels of HIF-1α, HK2, NLRP3, ASC, and Caspase-1 in the cartilage tissues from the KOA group were markedly higher compared to the Control group (Figures 6A,B). Following treatment with AP and HAP, the expression levels of these proteins were significantly decreased, with a more notable reduction in the HAP group (Figures 6C–G). Collectively, the results indicate that HAP mitigates KOA damage by inhibiting glycolysis and NLRP3 inflammasome activation.

### HAP suppresses the expression of proteins related to autophagy and apoptosis

**FIGURE 7:**
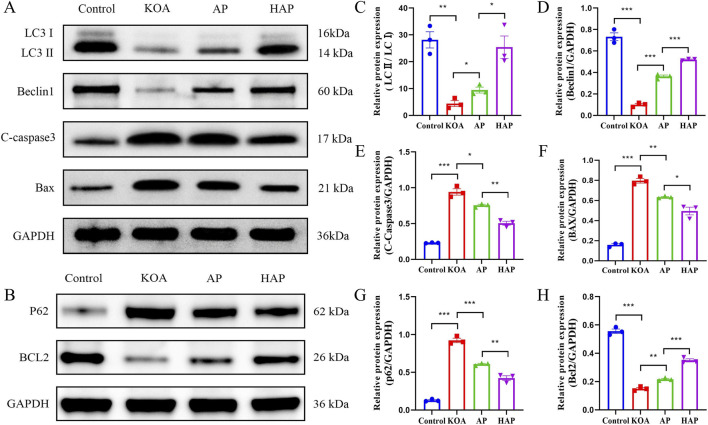
Effects of HAP on cartilage autophagy and apoptosis in KOA model rats. Note: **(A,C–F)** Expression of LC3 I, LC3 II, Beclin1, C-Caspase3 and Bax in cartilage tissue (GAPDH as internal control). **(B,G,H)** Expression of p62 and Bcl2 in cartilage (GAPDH as internal control). ***p < 0.001, **p < 0.01, *p < 0.05.

Autophagy in chondrocytes is crucial during the pathogenesis of OA, engaging in cellular metabolism, inflammation, apoptosis, and senescence, and is essential for the maintenance of intracellular homeostasis (Li L. et al., 2024). Activation of autophagy helps to inhibit chondrocyte apoptosis, thereby reducing joint inflammation and damage (Lee et al., 2024). Consequently, regulating chondrocyte autophagy and apoptosis offers novel approaches for the prevention and treatment of OA. Previous studies have demonstrated that acupuncture has a beneficial regulatory effect on autophagy, capable of both enhancing autophagy to remove damaged cellular structures and inhibiting autophagy to alleviate damage (Zhao et al., 2023). Western blot analysis in this study revealed that the protein expression levels of LC3-II, Beclin-1, and Bcl-2 in cartilage tissue from the KOA group were significantly decreased, while the expression of C-caspase3, Bax, and p62 proteins was markedly increased, suggesting that autophagy in chondrocytes was suppressed and the level of apoptosis was enhanced (Figures 7A,B). After intervention with AP and HAP, the expression levels of the aforementioned proteins significantly rebounded, with a more pronounced trend in the HAP group (Figures 7C–H). In summary, the results indicate that HAP can activate autophagy in chondrocytes and inhibit apoptosis.

## Discussion

With the acceleration of population aging, the incidence of KOA is gradually increasing, and it is projected that by 2050, the number of global KOA patients will reach 642 million, while currently there is a lack of effective management plans (GBD, 2021 Osteoarthritis Collaborators, 2023; Veronese et al., 2022). Current first-line non-surgical treatments such as NSAIDs primarily alleviate joint pain and improve functional activities by inhibiting the release of inflammatory factors, yet they fail to impede the pathological process of OA, and prolonged use could potentially intensify symptoms (Salis and Sainsbury, 2024). Furthermore, there is a significant discrepancy between the recommended treatment protocols in OA guidelines and actual clinical treatments in the real world. The constraints of various treatment options underscore the need to explore alternative therapies for OA management (Langworthy et al., 2024). Acupuncture has achieved satisfactory therapeutic effects in analgesia, especially in relieving pain and improving functional mobility (Tu et al., 2024; Zhu L. et al., 2024; Zhao et al., 2024). Clinical practice and guidelines demonstrate that acupuncture for KOA can reduce patient pain and functional impairments, frequently being employed as an alternative treatment for the disease (Luo et al., 2023; Wang et al., 2021). HAP based on conventional acupuncture points and combined with Jiaji points, has achieved certain clinical efficacy in the treatment of KOA (Huang et al., 2022). Despite this, there is a lack of biological evidence supporting its effectiveness. Therefore, this study is the first to assess the potential of homologous point acupuncture in treating KOA through basic research methods and to elucidate its anti-inflammatory and analgesic mechanisms. The ultimate goal is to provide feasible or alternative treatment options for KOA and to promote functional joint healing.

In this study, we established a KOA rat model with complete inflammatory responses and joint damage using intra-articular injection of MIA. Subsequently, we evaluated the effects of HAP intervention on KOA. Our results revealed that both HAP and conventional acupuncture significantly reduced serum levels of inflammatory factors and lactate dehydrogenase in rat models of KOA, thereby exerting beneficial effects on knee joint damage. On the foundation of uncovering the anti-inflammatory effects of HAP, we delved into serum metabolomics and cartilage tissue proteomics, finding that HAP can modulate the expression of metabolites and proteins associated with immune and metabolic pathways, shedding light on the potential immunometabolic mechanisms of the anti-inflammatory action of HAP. Subsequently, Western blot was used to detect proteins related to glycolysis, NLRP3 inflammasome, autophagy, and apoptosis, confirming that HAP significantly downregulated proteins associated with glycolysis and the NLRP3 inflammasome, including HIF-1α, HK2, NLRP3, ASC, and Caspase-1. Additionally, HAP significantly upregulated the protein expression of LC3, Beclin-1, and Bcl-2, and significantly downregulated C-caspase3, Bax, and p62, indicating that HAP activated autophagy and inhibited apoptosis. Thus, this study elucidates that HAP may mitigate KOA progression through multiple pathways, including modulating glycolysis, NLRP3 inflammasome activation, autophagy, and apoptosis, thereby providing experimental evidence for its therapeutic mechanism.

Arachidonic acid is an important fatty acid in the body that plays a significant role in inflammatory responses. This study indicates that HAP can inhibit Fc gamma R-mediated phagocytosis and Fc epsilon RI signaling pathways, thereby reducing the levels of Arachidonic acid. Fc gamma R-mediated phagocytosis (Gallo et al., 2010; Acharya et al., 2020) and Fc epsilon RI signaling pathways (Huang et al., 2020) play significant roles in inflammatory responses, being able to induce the production of pro-inflammatory cytokines, including Arachidonic acid, IL-1β, IL-8, and TNF-α, among others. Elevated levels of Arachidonic acid expression often predict the occurrence and progression of inflammatory responses and oxidative stress, and are considered a key mechanism in the treatment of osteoarthritis (Yan et al., 2023; Chen et al., 2023). 1-SAG is a diacylglycerol containing polyunsaturated fatty acids that can activate protein kinase C (PKC), leading to inflammatory responses and apoptosis (Wen et al., 2011). In light of the research findings, HAP may alleviate systemic inflammatory responses by reducing the expression levels of Arachidonic acid and 1-SAG in serum and inhibiting the release of IL-6, IL-1β, and TNF-α.

Based on chondrocyte tissue Astral-DIA proteomics, 687 DEPs were identified between the HAP and KOA groups. Bioinformatics analysis of the molecular functions and biological processes of DEPs showed significant enrichment in binding, catalytic activity, and metabolic processes, as well as immune system processes. Furthermore, in the KEGG pathway annotation and analysis, DEPs between the two groups (HAP vs. KOA) were significantly enriched in immune and metabolic related pathways. Therefore, we hypothesize that HAP exerts therapeutic effects by regulating immune and metabolic processes. Through Western blot analysis, we found that key glycolytic factors HIF-1α and HK2, as well as NLRP3 inflammasome-related proteins, were significantly reduced in the cartilage of rats in the HAP group. These findings suggest that inhibition of glycolytic processes and NLRP3 inflammasome activation may contribute to the observed therapeutic effects. However, it is important to emphasize that these are associative findings; without functional perturbation experiments (e.g., pharmacological inhibition of glycolysis or NLRP3 blockade), causal relationships cannot be established. LDH is one of the important enzymes in glycolysis, catalyzing the conversion of pyruvate to lactate while generating NAD+, thereby maintaining the glycolytic process (Chandel, 2021). Biochemical assays revealed a significant decrease in LDH activity in the serum of rats in the HAP group. Glycolysis, NLRP3 inflammasomes, autophagy, and apoptosis interact with each other in osteoarthritis. Glycolysis affects inflammatory responses by inhibiting the activation of the NLRP3 inflammasome (Stanton et al., 2024). In metabolic disorders, autophagy can suppress the activation of the NLRP3 inflammasome by inhibiting the production of ROS or promoting the degradation of inflammasome components such as NLRP3, caspase-1, and ASC (Lv et al., 2021). Autophagy is instrumental in preserving intracellular homeostasis, offering novel therapeutic approaches for OA through the modulation of chondrocyte autophagy and apoptosis (Chen Z. et al., 2024). A substantial amount of research confirms that enhancing chondrocyte autophagy can reduce the release of inflammatory factors and apoptosis, thereby alleviating cartilage damage and slowing the progression of OA (Cui et al., 2024; Kan et al., 2024; Li H. et al., 2024). Through Western blot analysis, we found that the pro-autophagic proteins (LC3 and Beclin1) and anti-apoptotic protein (Bcl-2) in the cartilage tissue of rats in the HAP group were significantly upregulated. The results suggest that HAP might safeguard chondrocytes by enhancing autophagy and suppressing apoptosis.

**FIGURE 8:**
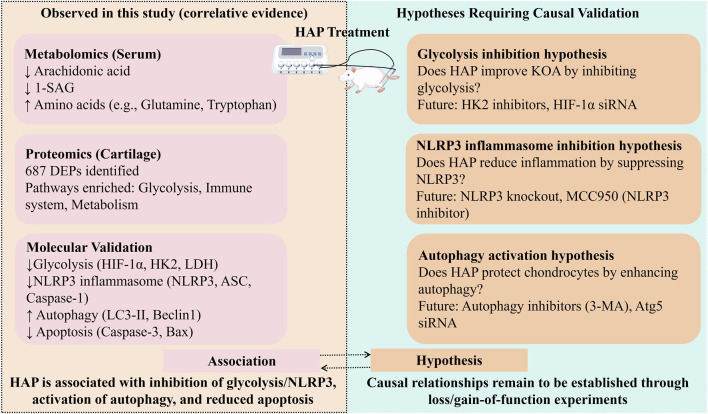
Schematic summary distinguishing observed associations from hypotheses requiring causal validation. Note: This schematic illustrates the two levels of evidence presented in this study. Left panel (Observed Associations): Findings derived from multi-omics and molecular analyses that demonstrate correlations between HAP treatment and changes in key biological pathways. Right panel (Hypotheses Requiring Causal Validation): Proposed mechanisms inferred from the associative data that require future functional perturbation experiments (e.g., pharmacological inhibition, genetic knockout) to establish causality.

This multi-omics study provides valuable insights into the mechanisms of HAP for treating KOA. However, several limitations should be acknowledged. From a methodological standpoint, the most significant limitation is the absence of a sham acupuncture control group. This design gap fundamentally constrains the interpretation of our findings: it is impossible to dissociate the specific therapeutic effects of HAP from the non-specific effects of the needling procedure itself (e.g., minor tissue trauma, restraint stress, or handling-induced analgesia). Consequently, all mechanistic interpretations presented in this study should be regarded as associative rather than causal (Figure 8). The observed changes in glycolysis, NLRP3 inflammasome, autophagy, and apoptosis pathways are correlated with HAP treatment, but whether these changes are specifically attributable to the unique acupoint selection strategy of HAP, or simply reflect generic needling responses, remains to be determined. Future studies incorporating a sham-controlled design (e.g., non-acupoint superficial insertion or non-penetrating sham needles) are essential to establish treatment specificity. Furthermore, model validation could be strengthened. More rigorous assessment of post-modeling behavioral changes (including lameness, joint swelling, and von Frey testing) would provide more robust confirmation of successful KOA induction. Regarding the outcomes, while our metabolomic and proteomic analyses identified numerous altered pathways, the study lacks functional validation to establish their causal roles in the efficacy of HAP. Additionally, an integrative analysis linking the altered metabolites to corresponding protein changes is required for a deeper understanding. Although HAP exhibited a stronger therapeutic trend compared to conventional acupuncture, the biological basis for this superiority remains largely unexplored. A comparative multi-omics analysis between HAP and AP groups might reveal unique mechanisms specific to HAP. Therefore, future work should focus on the following directions: First, employing gain- and loss-of-function experiments (e.g., using pharmacological inhibitors or siRNA) is essential to causally link the top candidate pathways (e.g., glycolysis, NLRP3 inflammasome activation) to HAP’s therapeutic effects, thereby moving from correlation to causation. Second, the HAP concept requires anatomical and functional validation. Neural tract tracing studies to map the spinal segments targeted by HAP, combined with central nervous system imaging, could provide a scientific foundation for this TCM theory. Subsequent preclinical studies must also incorporate a sham control group to definitively establish treatment specificity. Finally, investigations into the mechanism of HAP should be expanded to encompass other critical OA pathological processes, such as cellular senescence and detailed extracellular matrix remodeling.

## Conclusion

In summary, our study found that HAP can effectively improve cartilage damage in a KOA rat model. Our research results reveal the potential mechanism of HAP in treating KOA; HAP may improve chondrocyte metabolic disorders and inflammatory responses by inhibiting glycolysis and NLRP3 inflammasome activation, and by promoting chondrocyte autophagy and reducing apoptosis levels, thereby exerting therapeutic effects. These results provide new therapeutic insights for the clinical treatment of KOA, especially in the pursuit of enhancing the efficacy of acupuncture.

## Data Availability

The raw data supporting the conclusions of this article will be made available by the authors, without undue reservation.
