# Supplementary figures and images for: Uncovering the mechanisms of homologous point acupuncture on knee osteoarthritis through an integrated study of metabolomics and proteomics
Source: Front Bioeng Biotechnol. 2026 Apr 22;14:1791109. doi: 10.3389/fbioe.2026.1791109 (PMC13147163; doi:10.3389/fbioe.2026.1791109)

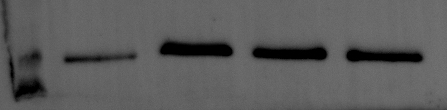

Supplement: Supplementary file 1 [file DataSheet1.zip › bax-1-Original picture.jpg]

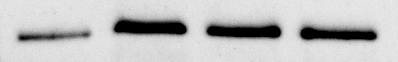

Supplement: Supplementary file 1 [file DataSheet1.zip › bax-1.jpg]

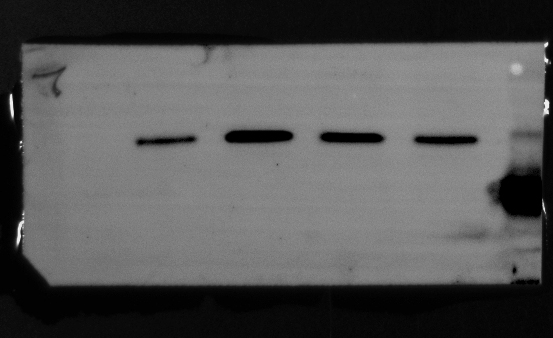

Supplement: Supplementary file 1 [file DataSheet1.zip › bax-2-Original picture.jpg]

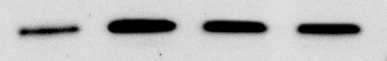

Supplement: Supplementary file 1 [file DataSheet1.zip › bax-2.jpg]

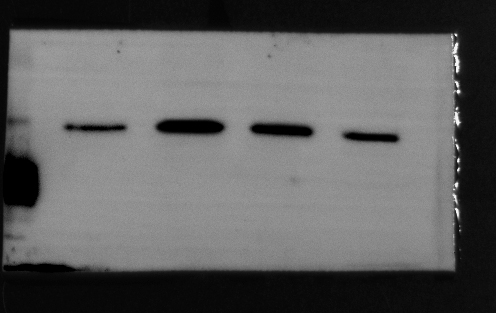

Supplement: Supplementary file 1 [file DataSheet1.zip › bax-3-Original picture.jpg]

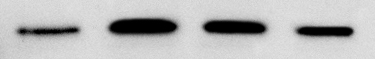

Supplement: Supplementary file 1 [file DataSheet1.zip › bax-3.jpg]

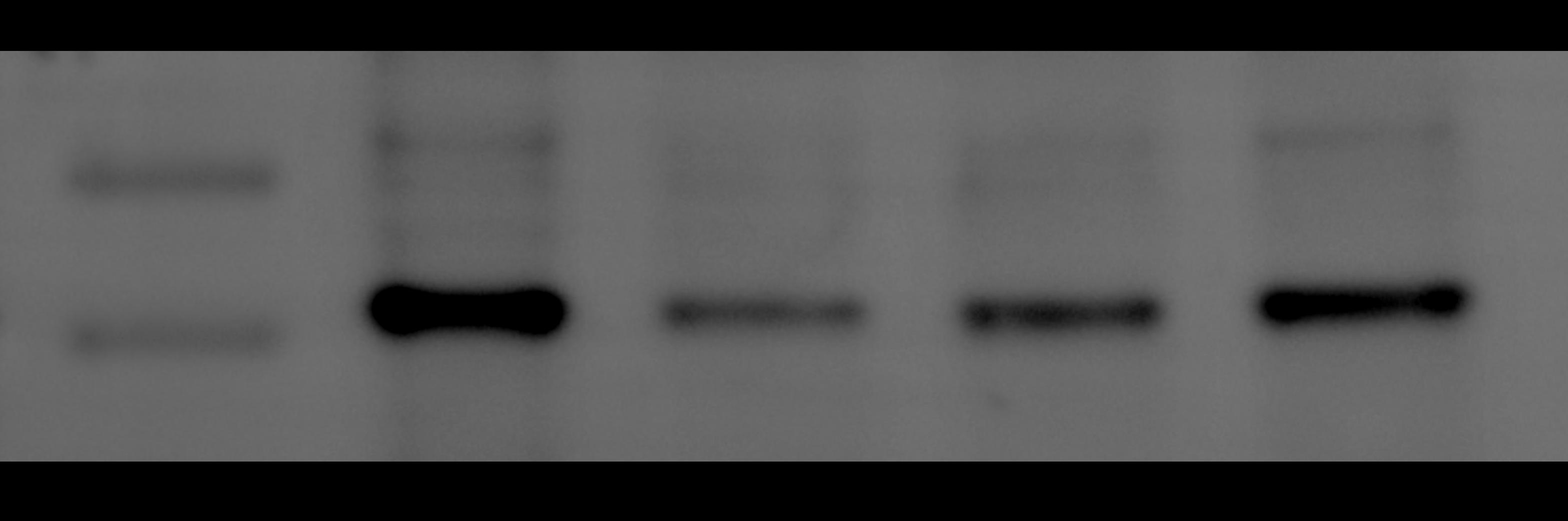

Supplement: Supplementary file 1 [file DataSheet1.zip › BCL2-1-Original picture.tiff]

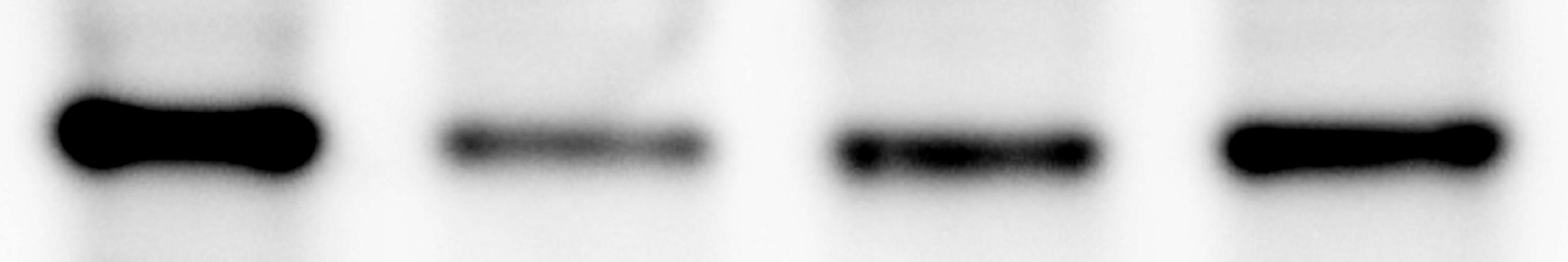

Supplement: Supplementary file 1 [file DataSheet1.zip › BCL2-1.jpg]

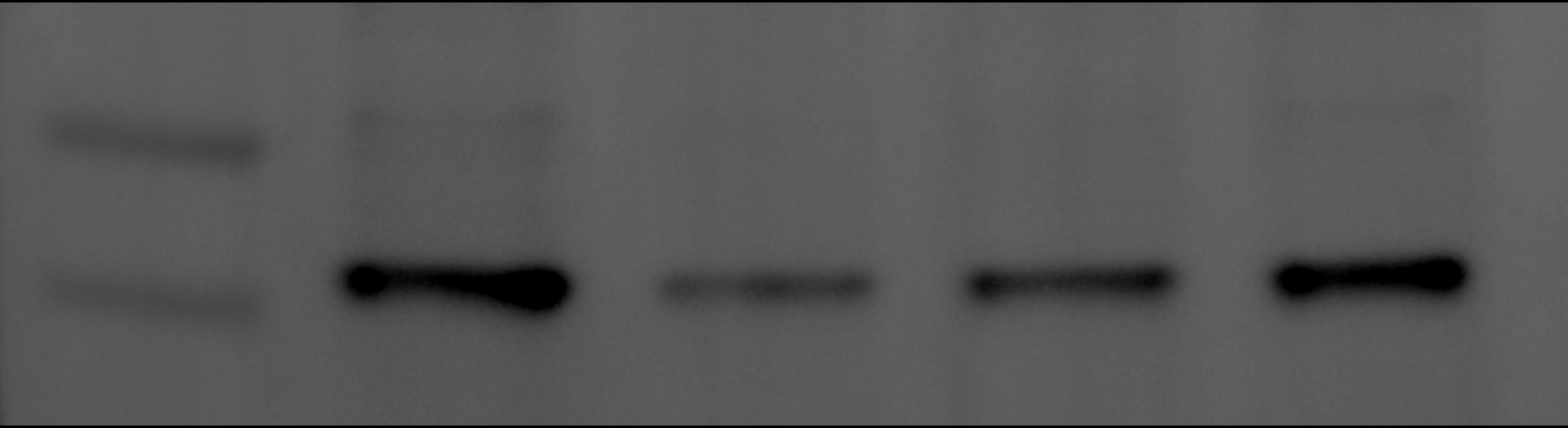

Supplement: Supplementary file 1 [file DataSheet1.zip › BCL2-2-Original picture.tiff]

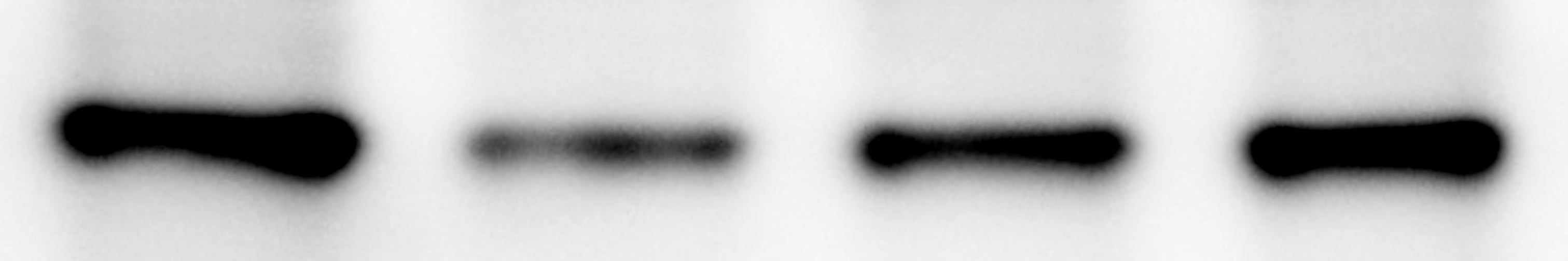

Supplement: Supplementary file 1 [file DataSheet1.zip › BCL2-2.jpg]

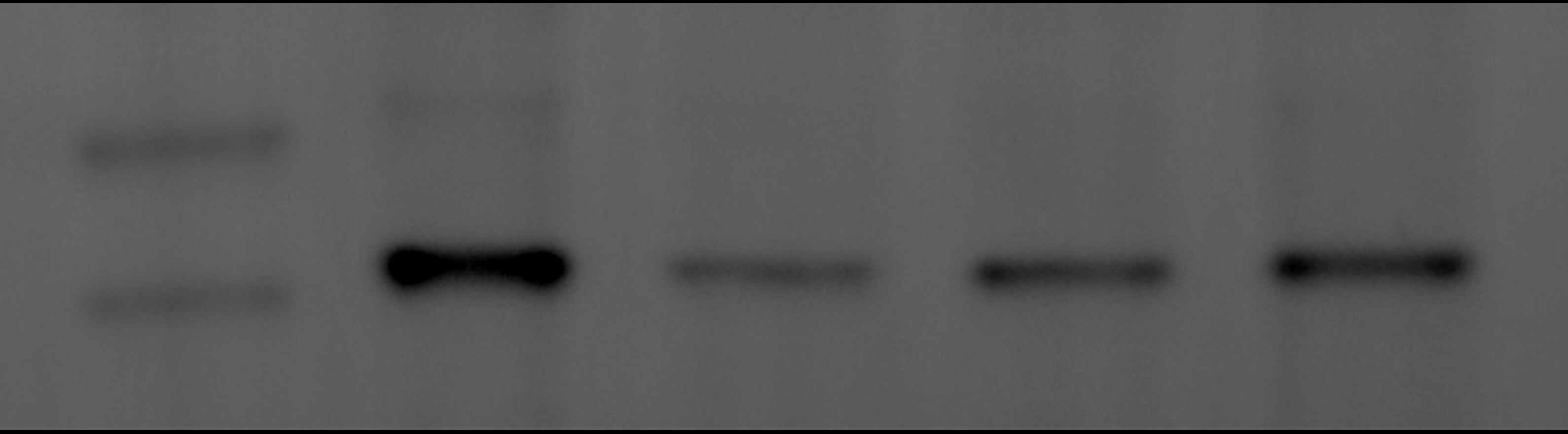

Supplement: Supplementary file 1 [file DataSheet1.zip › BCL2-3-Original picture.tiff]

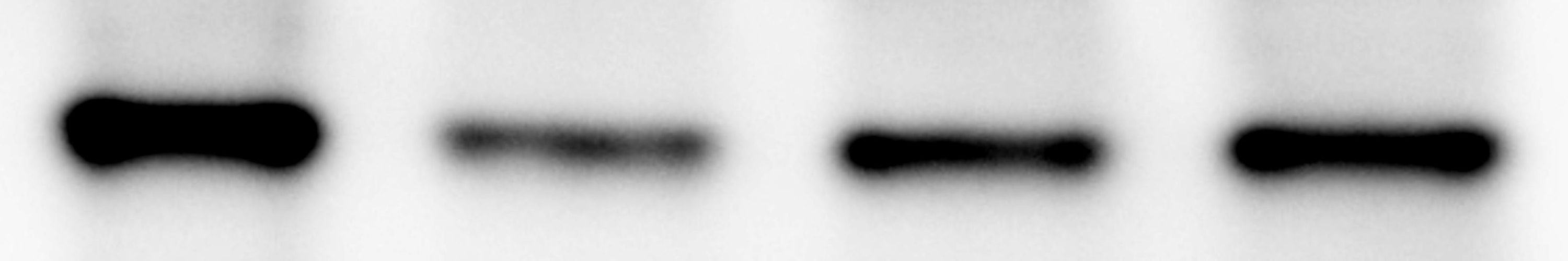

Supplement: Supplementary file 1 [file DataSheet1.zip › BCL2-3.jpg]

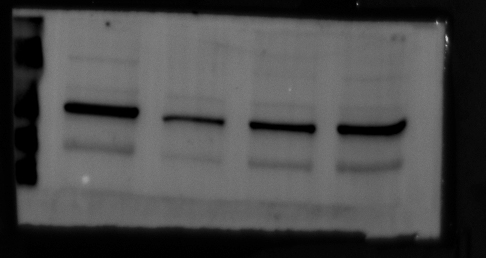

Supplement: Supplementary file 1 [file DataSheet1.zip › beclin1-1-Original picture.jpg]

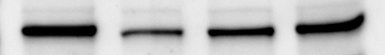

Supplement: Supplementary file 1 [file DataSheet1.zip › beclin1-1.jpg]

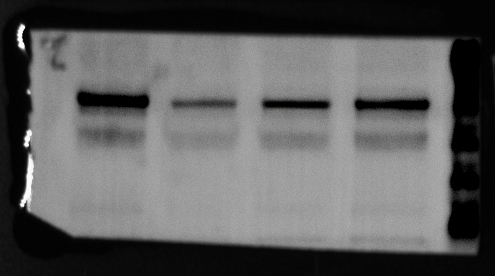

Supplement: Supplementary file 1 [file DataSheet1.zip › beclin1-2-Original picture.jpg]

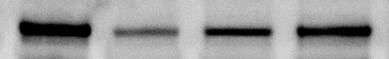

Supplement: Supplementary file 1 [file DataSheet1.zip › beclin1-2.jpg]

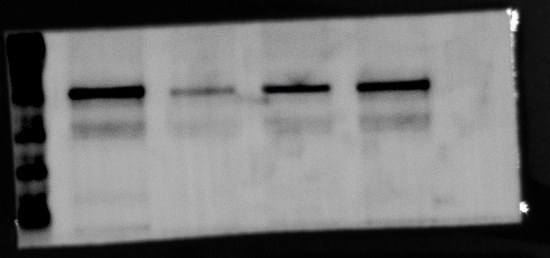

Supplement: Supplementary file 1 [file DataSheet1.zip › beclin1-3-Original picture.jpg]

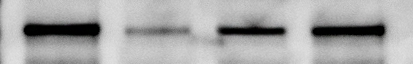

Supplement: Supplementary file 1 [file DataSheet1.zip › beclin1-3.jpg]

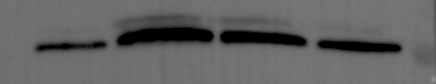

Supplement: Supplementary file 1 [file DataSheet1.zip › C-caspase3-1-Original picture.jpg]

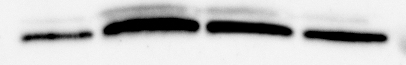

Supplement: Supplementary file 1 [file DataSheet1.zip › C-caspase3-1.jpg]

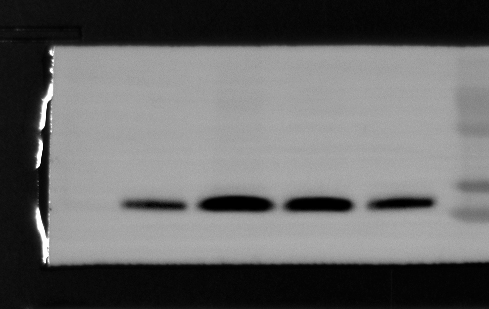

Supplement: Supplementary file 1 [file DataSheet1.zip › C-caspase3-2-Original picture.jpg]

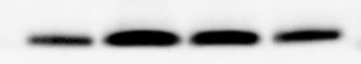

Supplement: Supplementary file 1 [file DataSheet1.zip › C-caspase3-2.jpg]

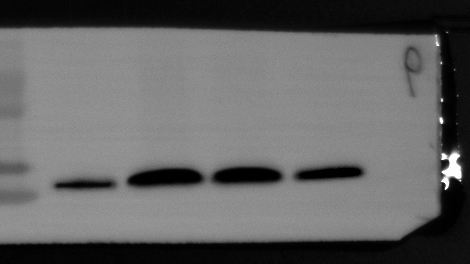

Supplement: Supplementary file 1 [file DataSheet1.zip › C-caspase3-3-Original picture.jpg]

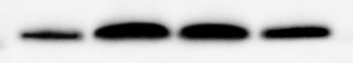

Supplement: Supplementary file 1 [file DataSheet1.zip › C-caspase3-3.jpg]

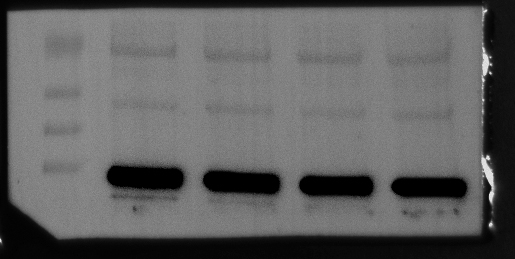

Supplement: Supplementary file 1 [file DataSheet1.zip › GAPDH-1-Original picture.jpg]

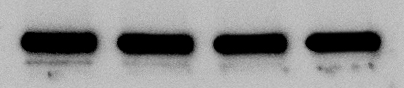

Supplement: Supplementary file 1 [file DataSheet1.zip › GAPDH-1.jpg]

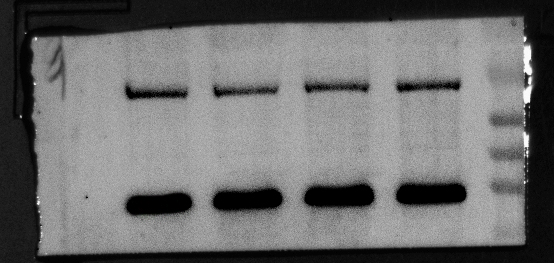

Supplement: Supplementary file 1 [file DataSheet1.zip › GAPDH-2-Original picture.jpg]

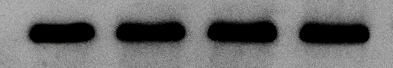

Supplement: Supplementary file 1 [file DataSheet1.zip › GAPDH-2.jpg]

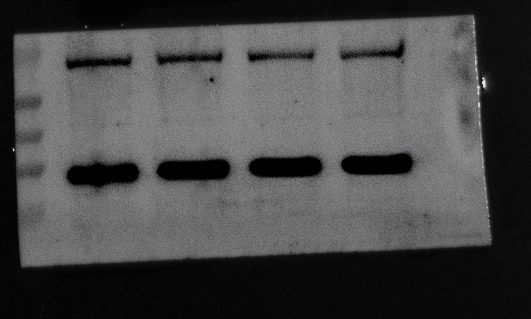

Supplement: Supplementary file 1 [file DataSheet1.zip › GAPDH-3-Original picture.jpg]

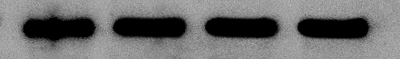

Supplement: Supplementary file 1 [file DataSheet1.zip › GAPDH-3.jpg]

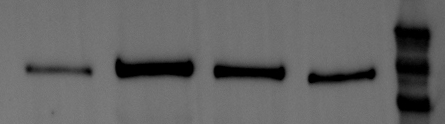

Supplement: Supplementary file 1 [file DataSheet1.zip › HIF1α-1-Original picture.jpg]

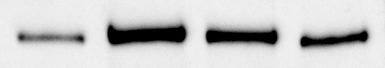

Supplement: Supplementary file 1 [file DataSheet1.zip › HIF1α-1.jpg]

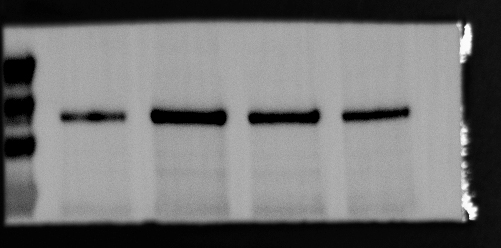

Supplement: Supplementary file 1 [file DataSheet1.zip › HIF1α-2-Original picture.jpg]

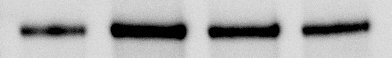

Supplement: Supplementary file 1 [file DataSheet1.zip › HIF1α-2.jpg]

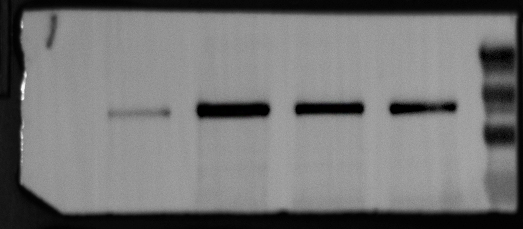

Supplement: Supplementary file 1 [file DataSheet1.zip › HIF1α-3-Original picture.jpg]

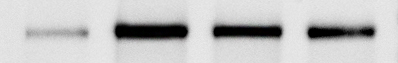

Supplement: Supplementary file 1 [file DataSheet1.zip › HIF1α-3.jpg]

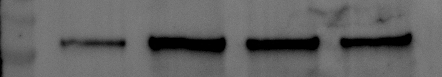

Supplement: Supplementary file 1 [file DataSheet1.zip › HK2-1-Original picture.jpg]

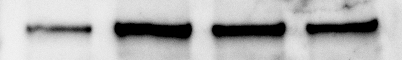

Supplement: Supplementary file 1 [file DataSheet1.zip › HK2-1.jpg]

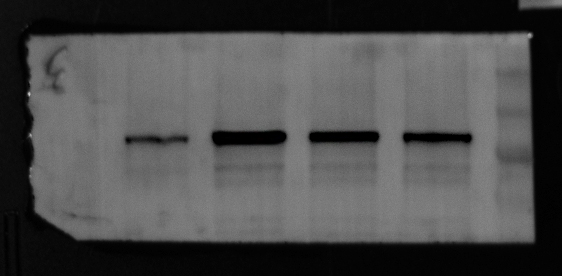

Supplement: Supplementary file 1 [file DataSheet1.zip › HK2-2-Original picture.jpg]

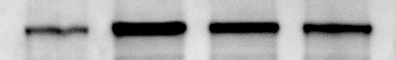

Supplement: Supplementary file 1 [file DataSheet1.zip › HK2-2.jpg]

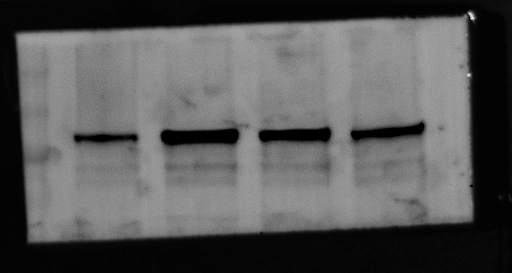

Supplement: Supplementary file 1 [file DataSheet1.zip › HK2-3-Original picture.jpg]

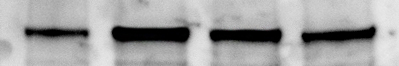

Supplement: Supplementary file 1 [file DataSheet1.zip › HK2-3.jpg]

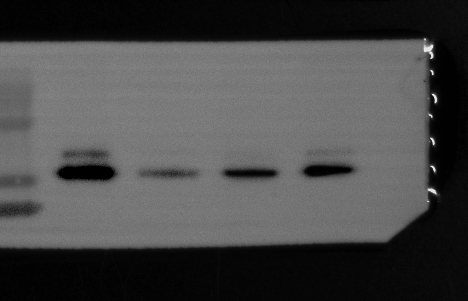

Supplement: Supplementary file 1 [file DataSheet1.zip › LC3-1-Original picture.jpg]

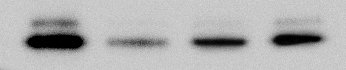

Supplement: Supplementary file 1 [file DataSheet1.zip › LC3-1.jpg]

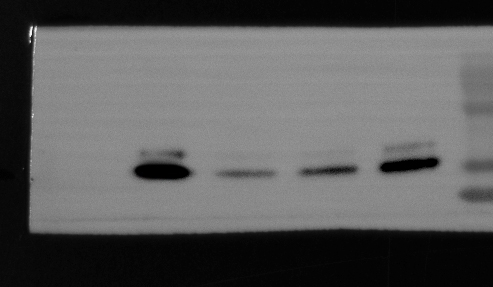

Supplement: Supplementary file 1 [file DataSheet1.zip › LC3-2-Original picture.jpg]

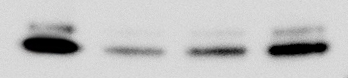

Supplement: Supplementary file 1 [file DataSheet1.zip › LC3-2.jpg]

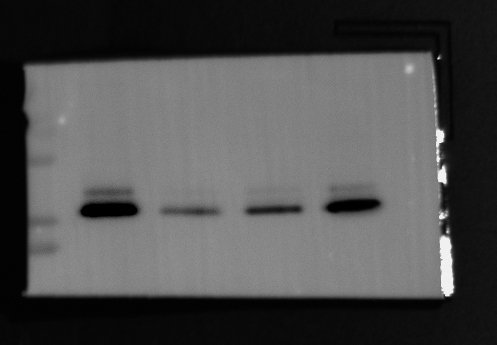

Supplement: Supplementary file 1 [file DataSheet1.zip › LC3-3-Original picture.jpg]

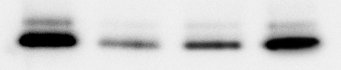

Supplement: Supplementary file 1 [file DataSheet1.zip › LC3-3.jpg]

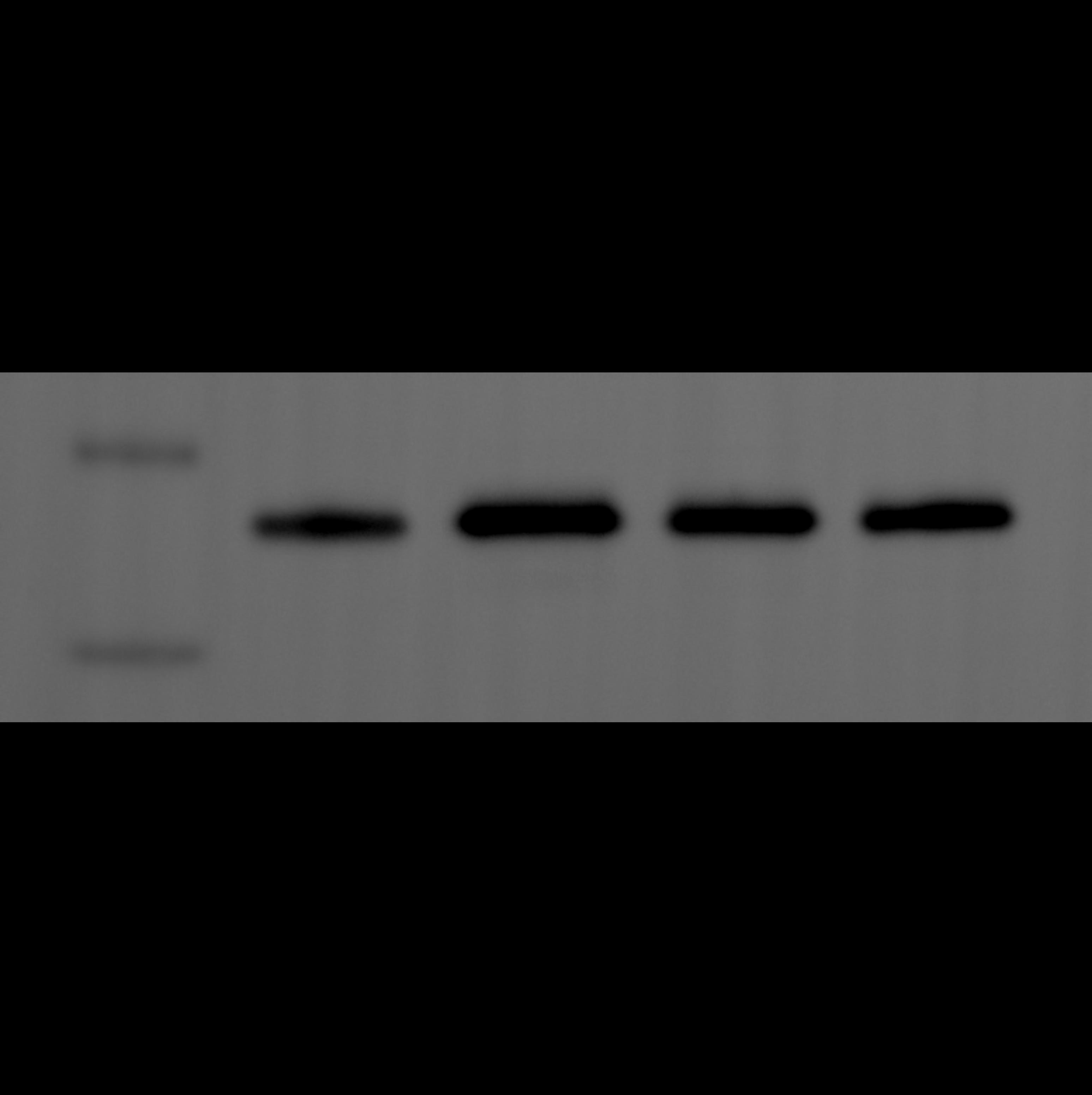

Supplement: Supplementary file 1 [file DataSheet1.zip › NLRP3/ASC-1-Original picture.tiff]

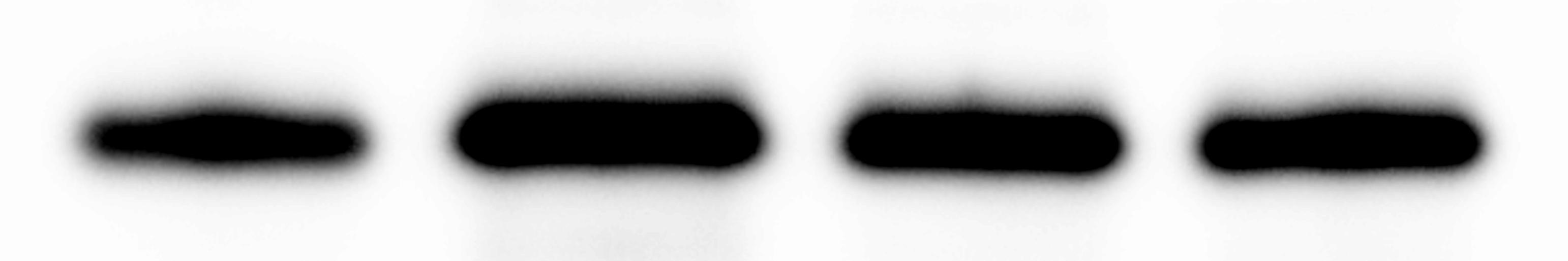

Supplement: Supplementary file 1 [file DataSheet1.zip › NLRP3/ASC-1.jpg]

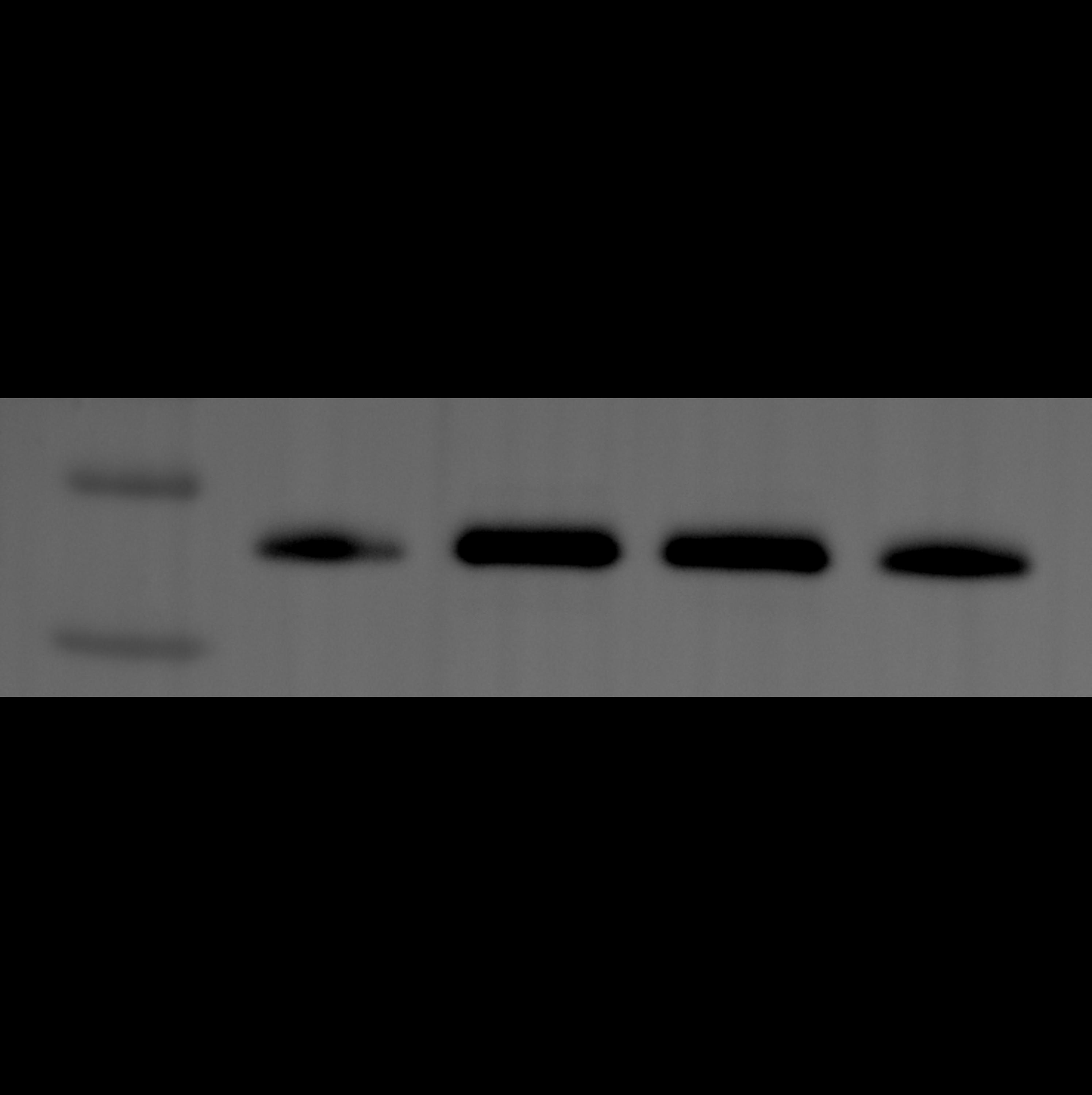

Supplement: Supplementary file 1 [file DataSheet1.zip › NLRP3/ASC-2-Original picture.tiff]

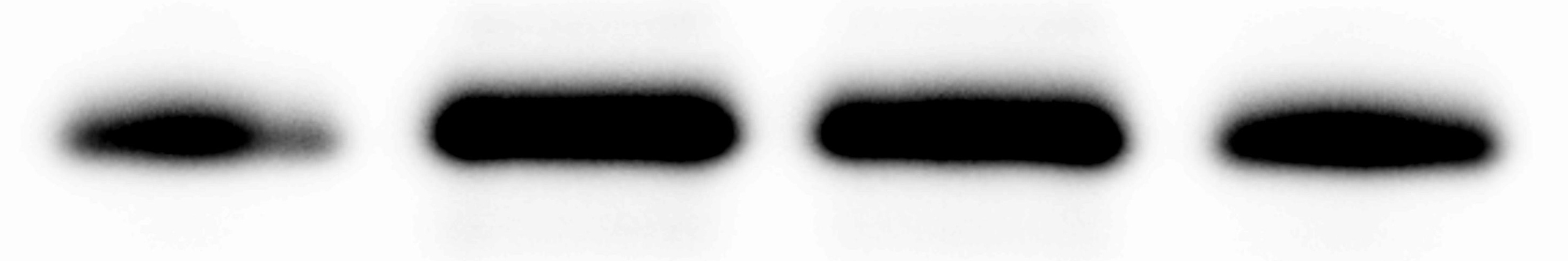

Supplement: Supplementary file 1 [file DataSheet1.zip › NLRP3/ASC-2.jpg]

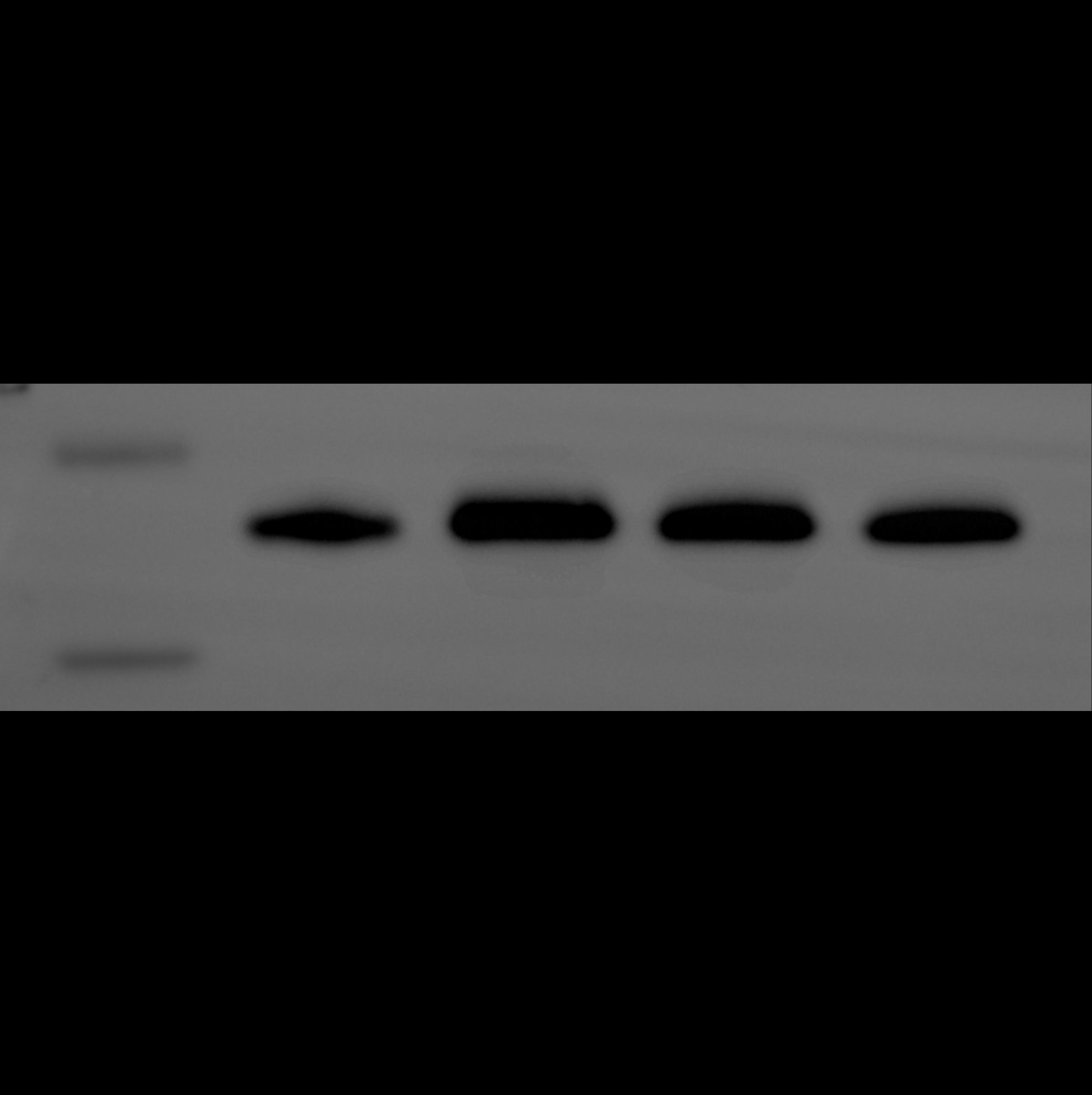

Supplement: Supplementary file 1 [file DataSheet1.zip › NLRP3/ASC-3-Original picture.tiff]

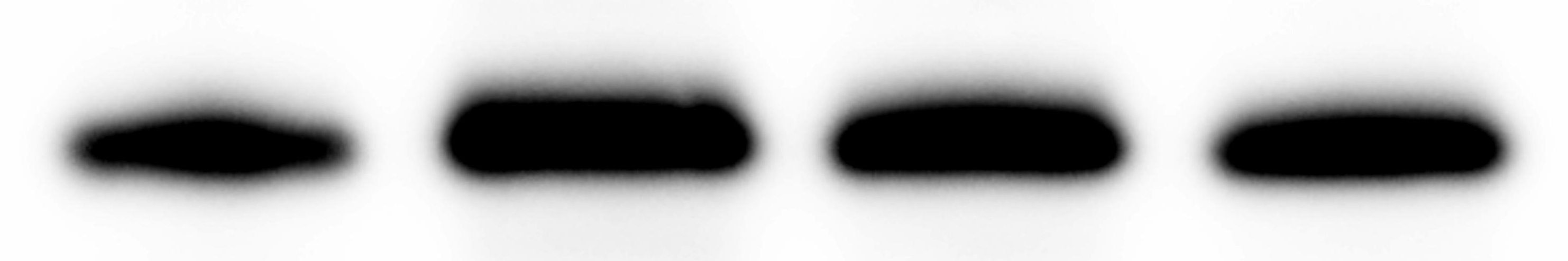

Supplement: Supplementary file 1 [file DataSheet1.zip › NLRP3/ASC-3.jpg]

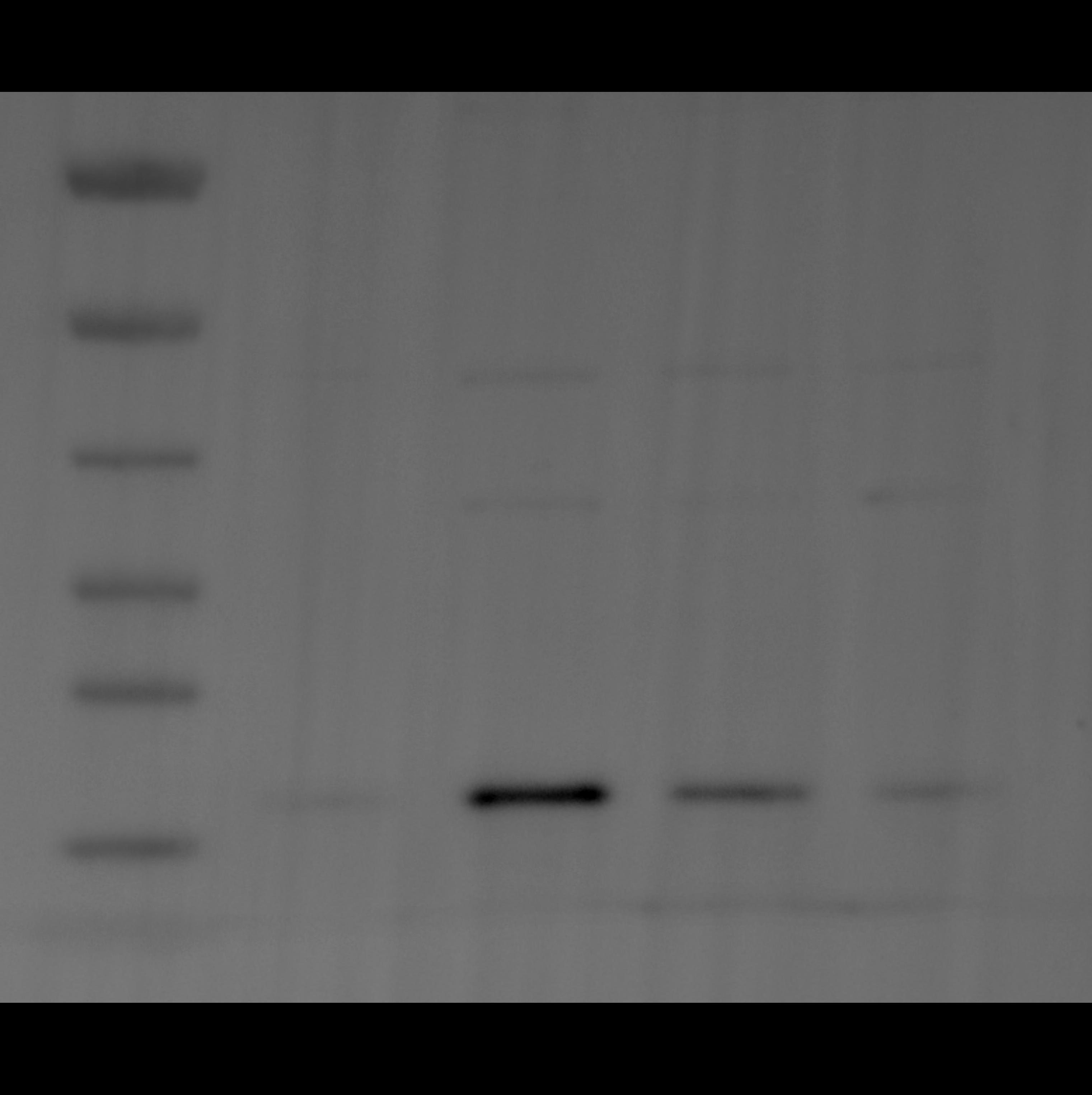

Supplement: Supplementary file 1 [file DataSheet1.zip › NLRP3/caspae1-1-Original picture.tiff]

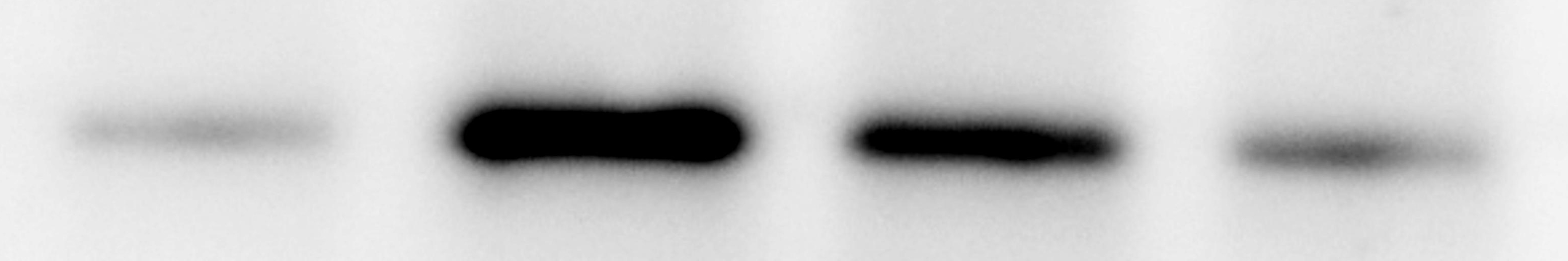

Supplement: Supplementary file 1 [file DataSheet1.zip › NLRP3/caspae1-1.jpg]

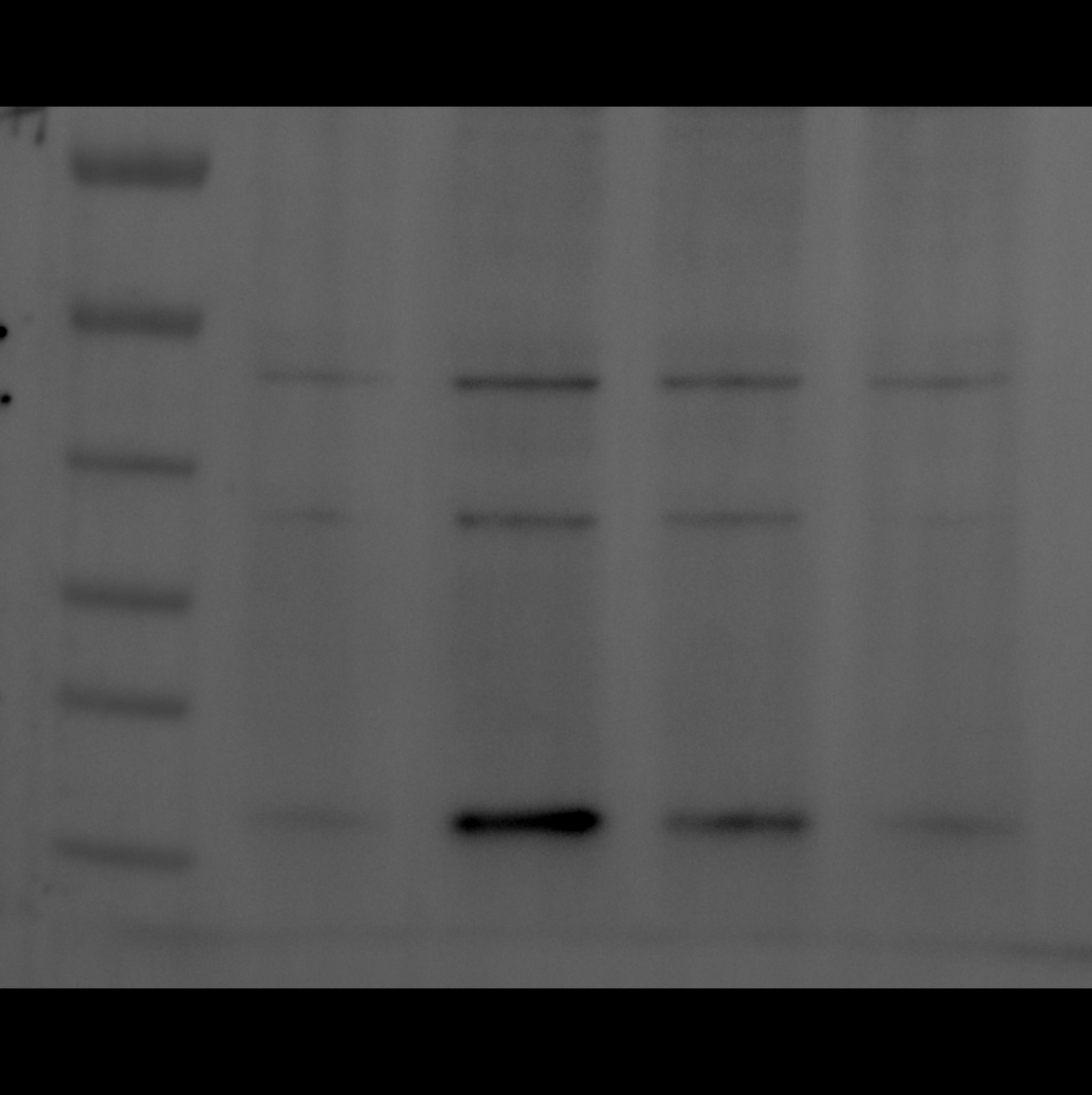

Supplement: Supplementary file 1 [file DataSheet1.zip › NLRP3/caspae1-2-Original picture.tiff]

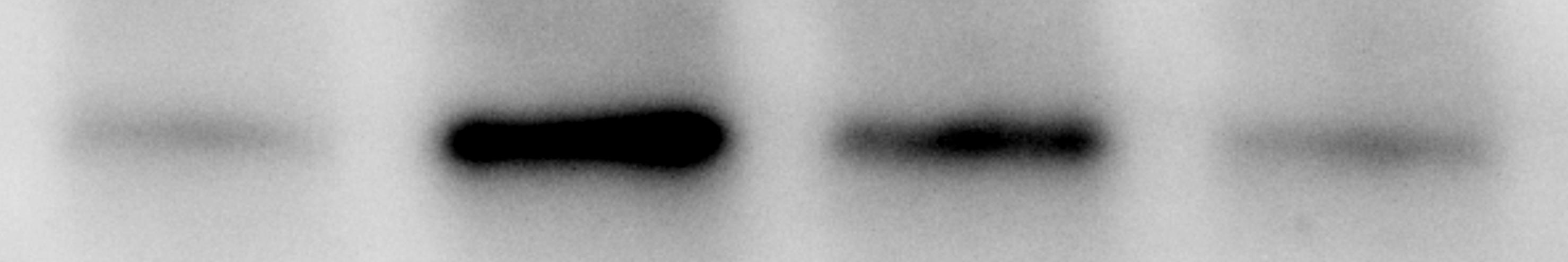

Supplement: Supplementary file 1 [file DataSheet1.zip › NLRP3/caspae1-2.jpg]

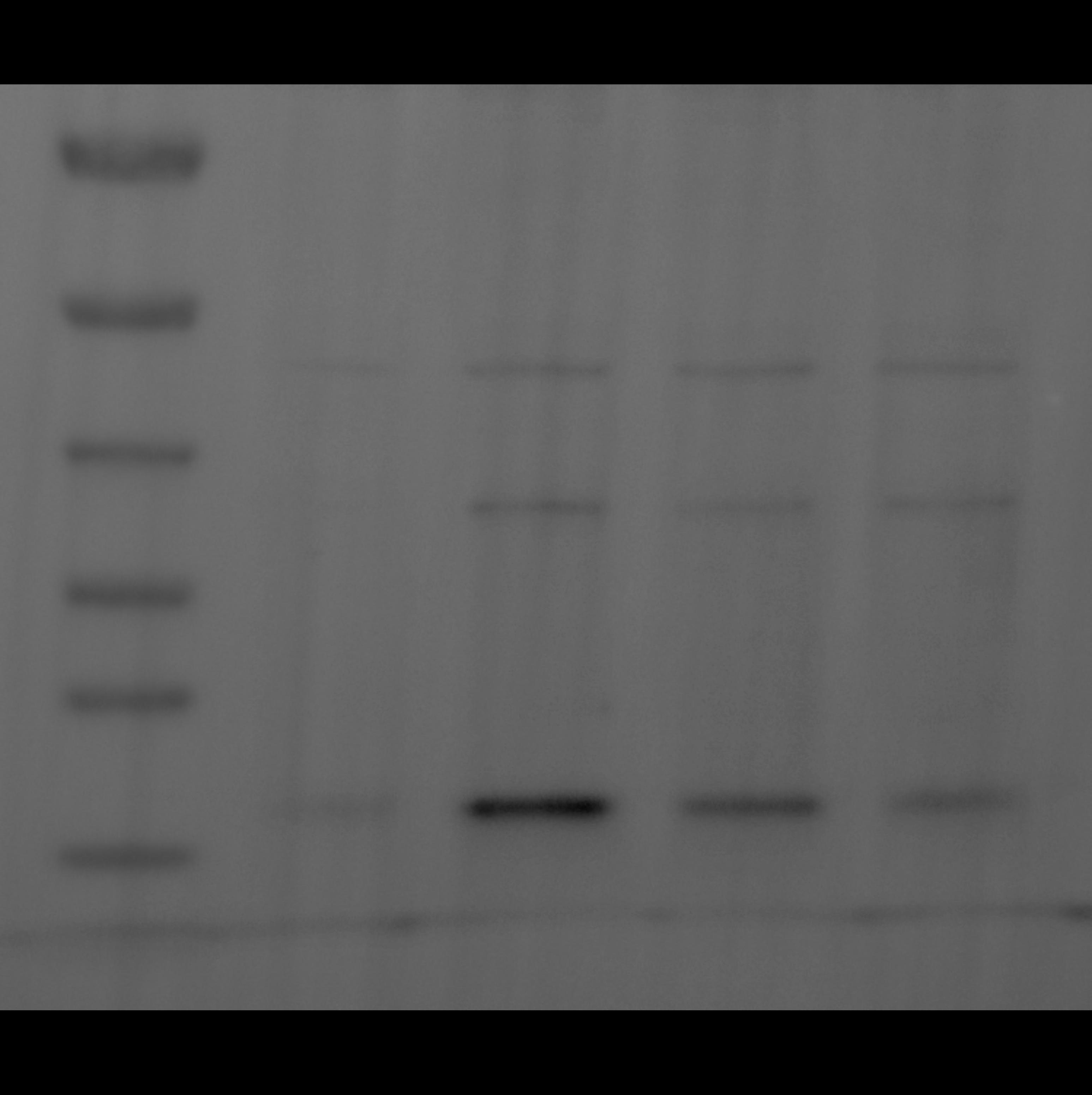

Supplement: Supplementary file 1 [file DataSheet1.zip › NLRP3/caspae1-3-Original picture.tiff]

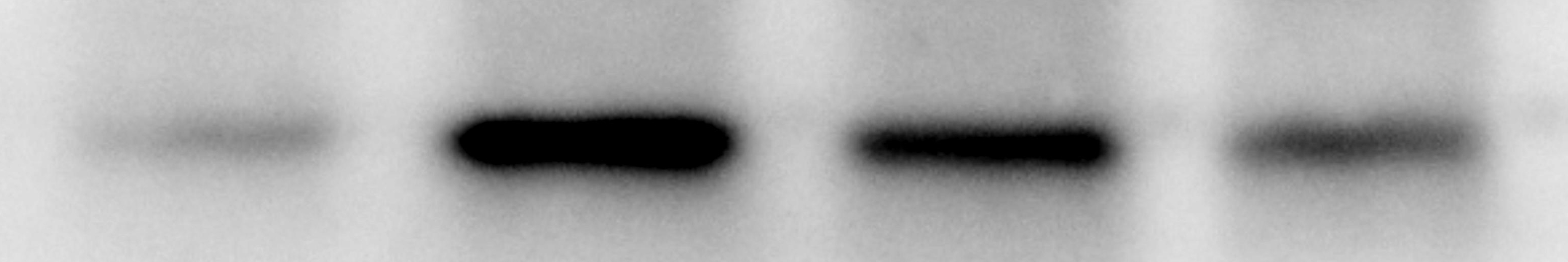

Supplement: Supplementary file 1 [file DataSheet1.zip › NLRP3/caspae1-3.jpg]

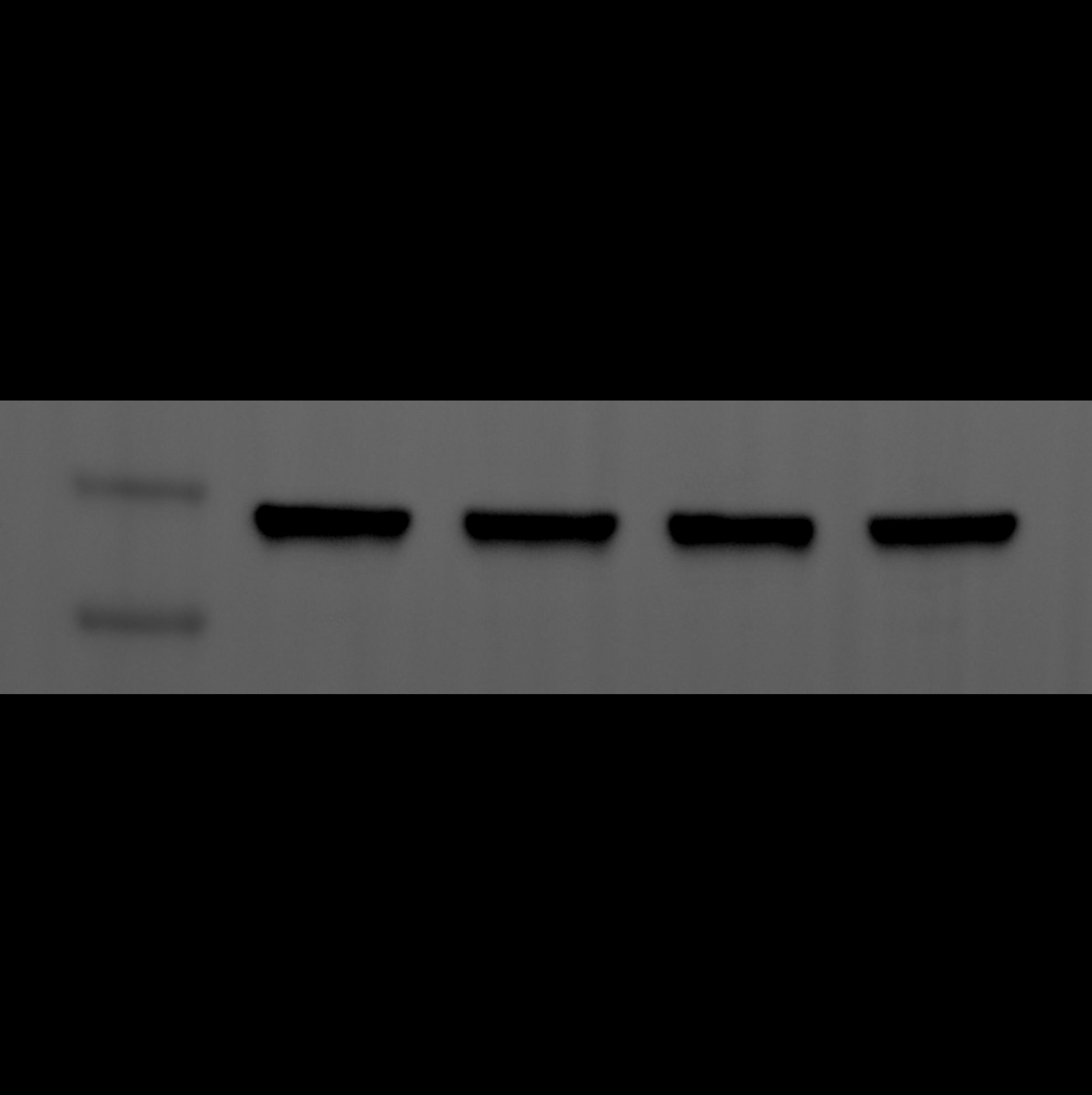

Supplement: Supplementary file 1 [file DataSheet1.zip › NLRP3/GAPDH-1-Original picture.tiff]

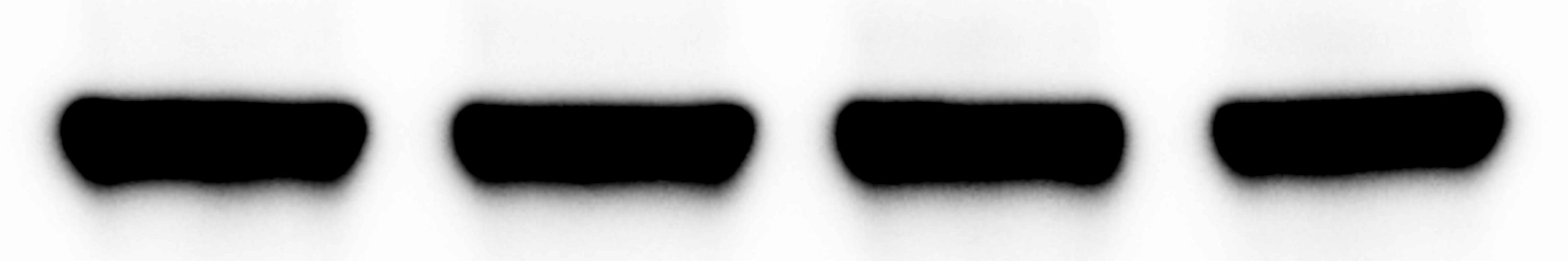

Supplement: Supplementary file 1 [file DataSheet1.zip › NLRP3/GAPDH-1.jpg]

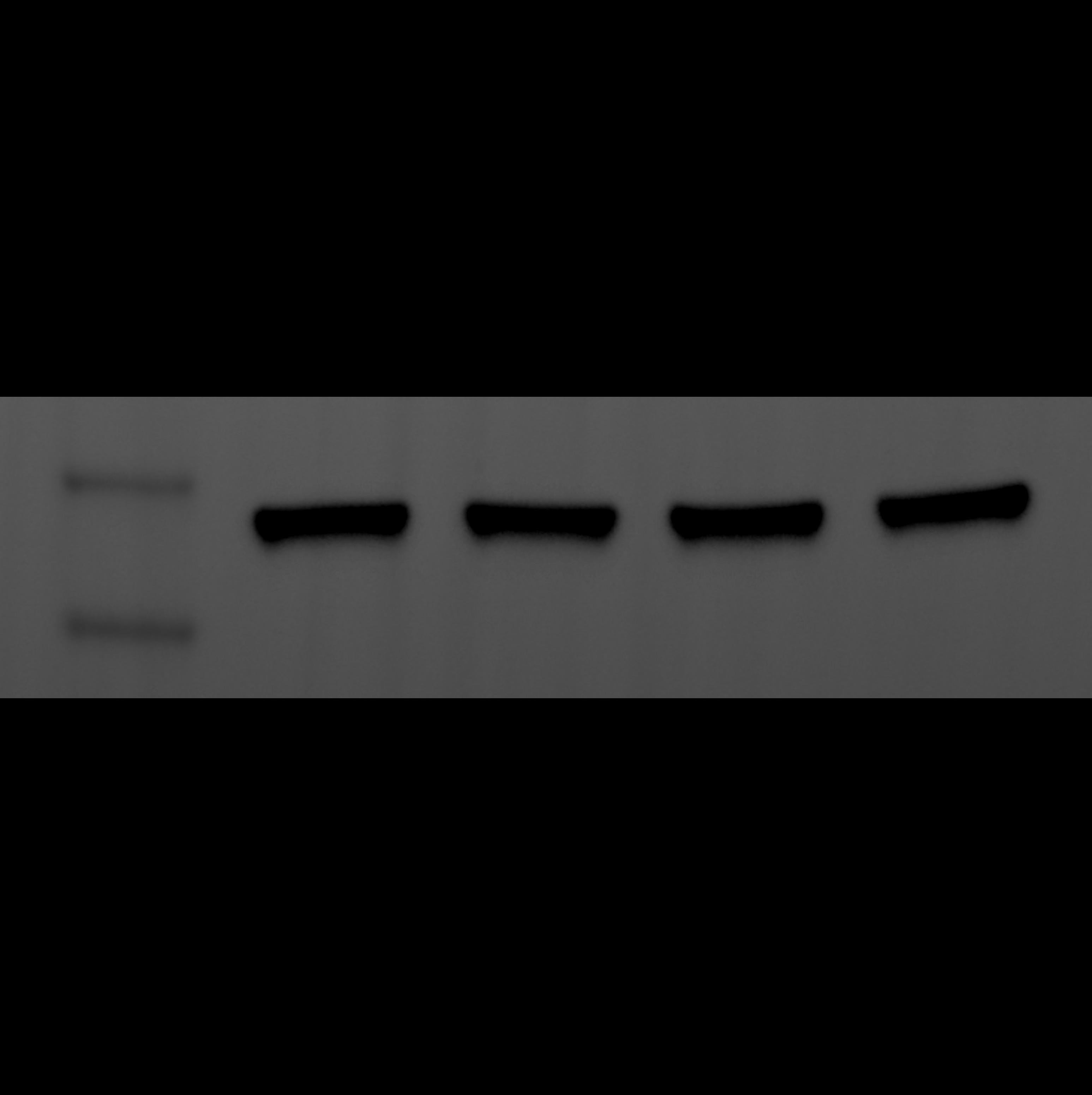

Supplement: Supplementary file 1 [file DataSheet1.zip › NLRP3/GAPDH-2-Original picture.tiff]

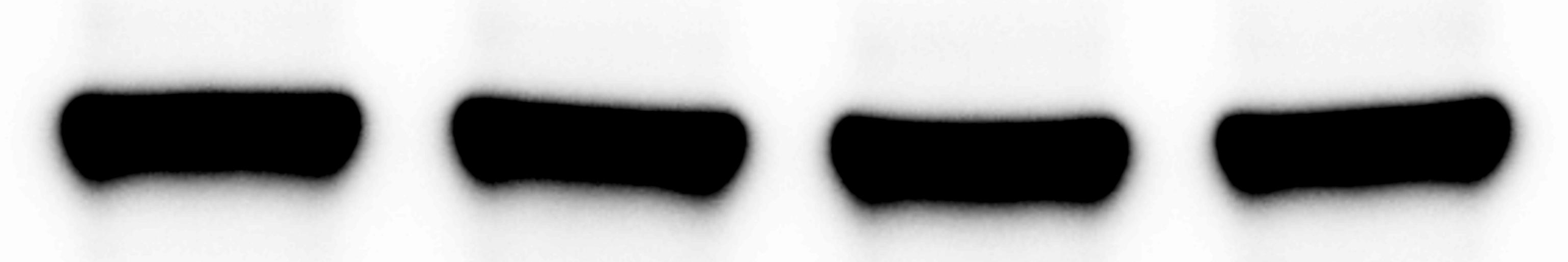

Supplement: Supplementary file 1 [file DataSheet1.zip › NLRP3/GAPDH-2.jpg]

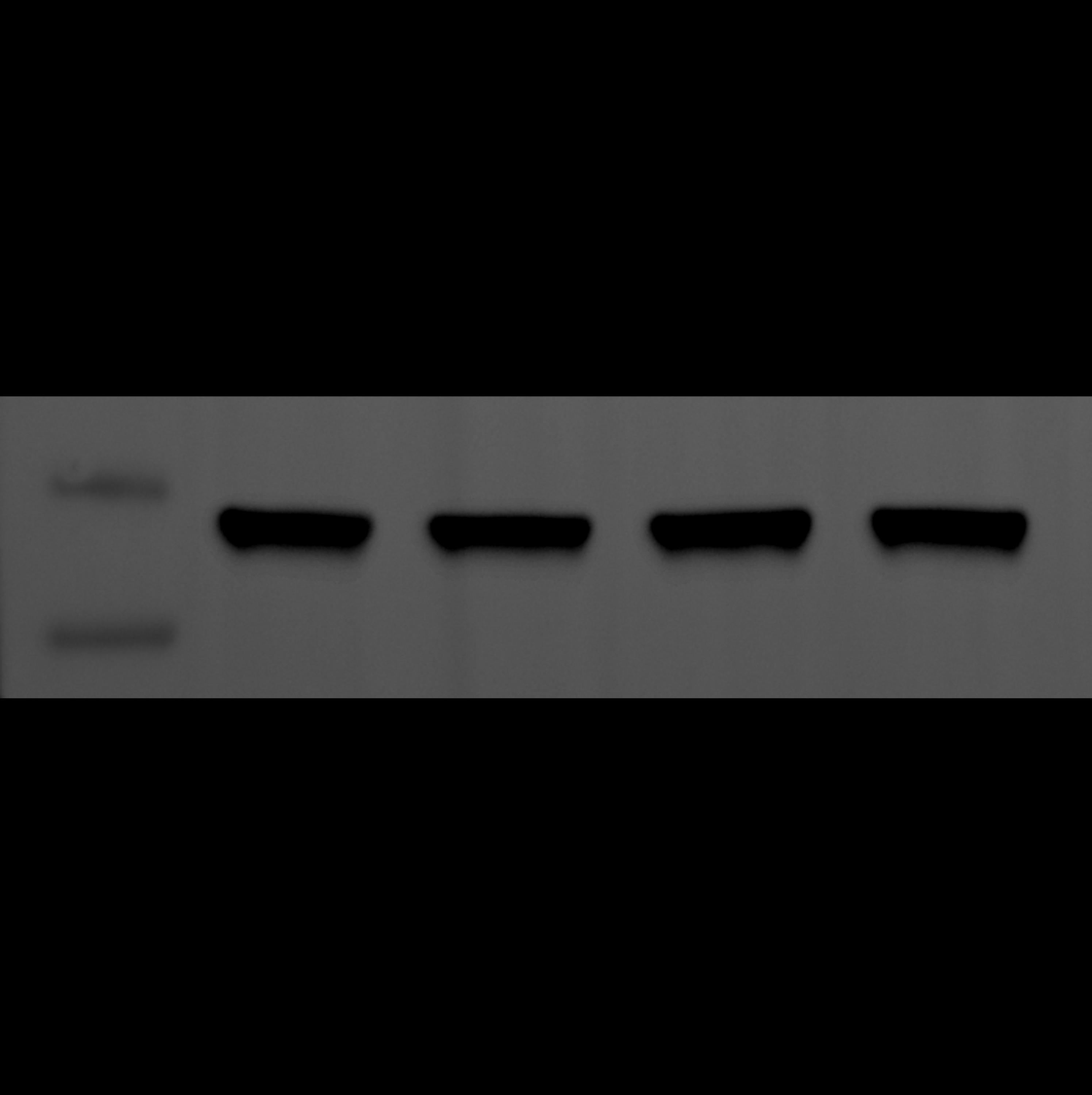

Supplement: Supplementary file 1 [file DataSheet1.zip › NLRP3/GAPDH-3-Original picture.tiff]

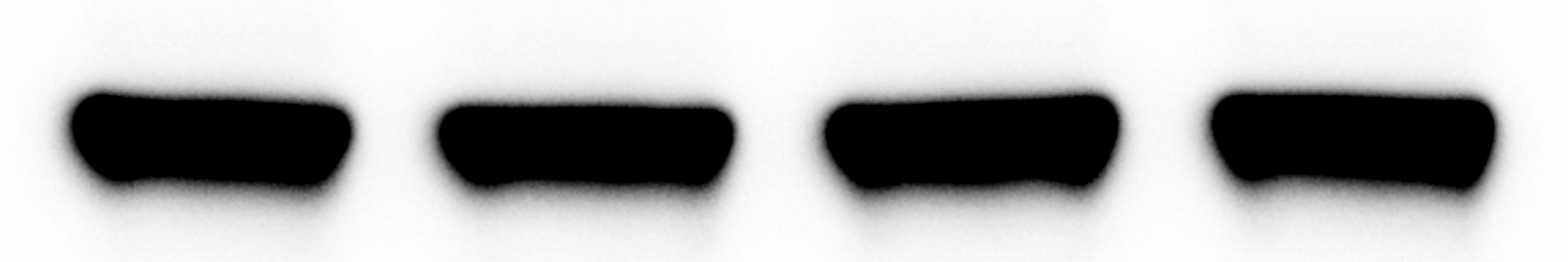

Supplement: Supplementary file 1 [file DataSheet1.zip › NLRP3/GAPDH-3.jpg]

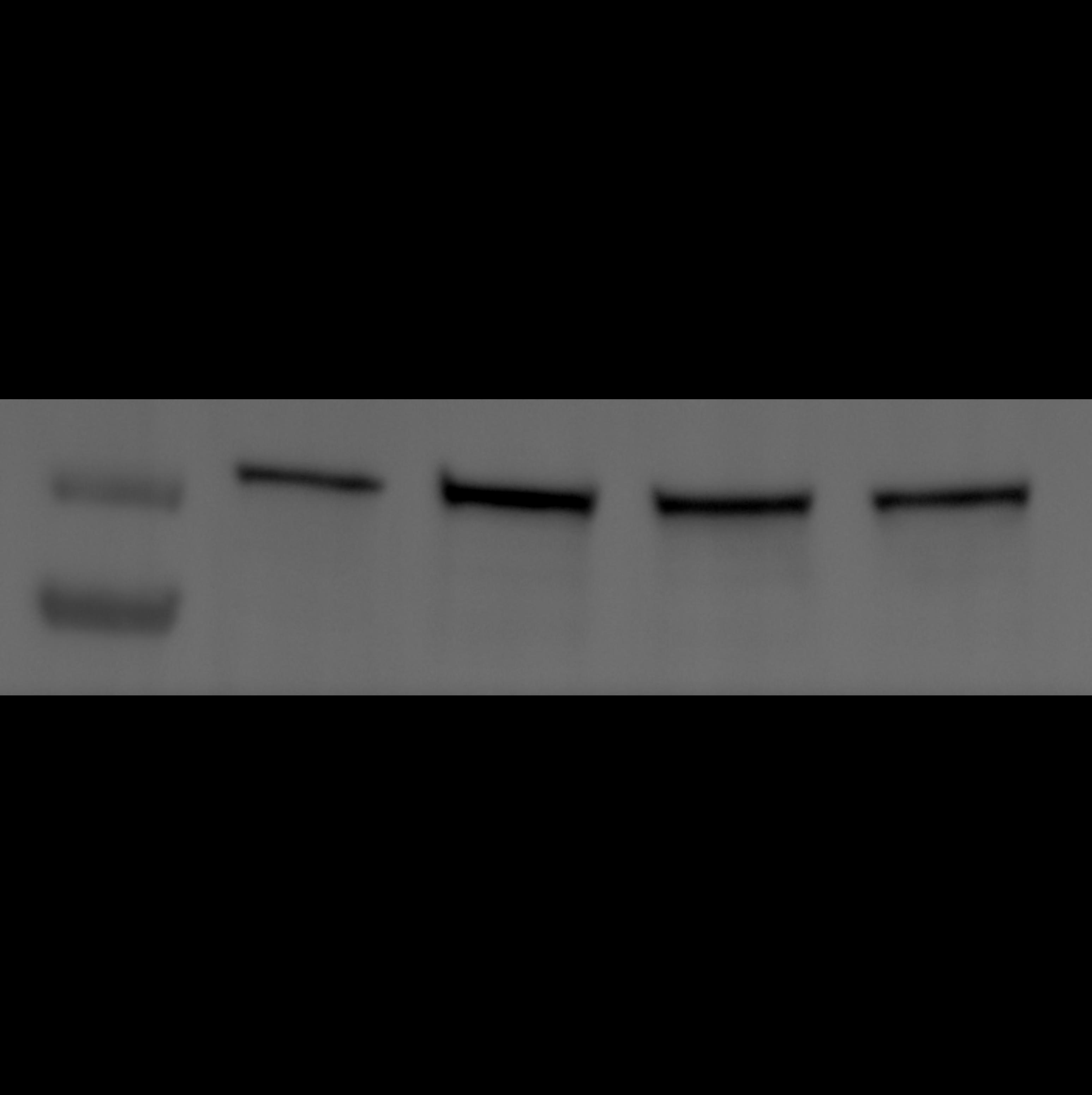

Supplement: Supplementary file 1 [file DataSheet1.zip › NLRP3/NLRP3-1-Original picture.tiff]

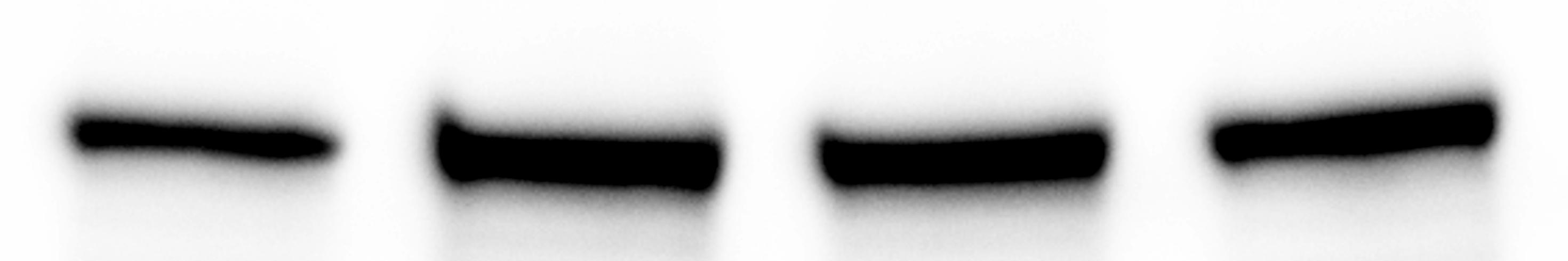

Supplement: Supplementary file 1 [file DataSheet1.zip › NLRP3/NLRP3-1.jpg]

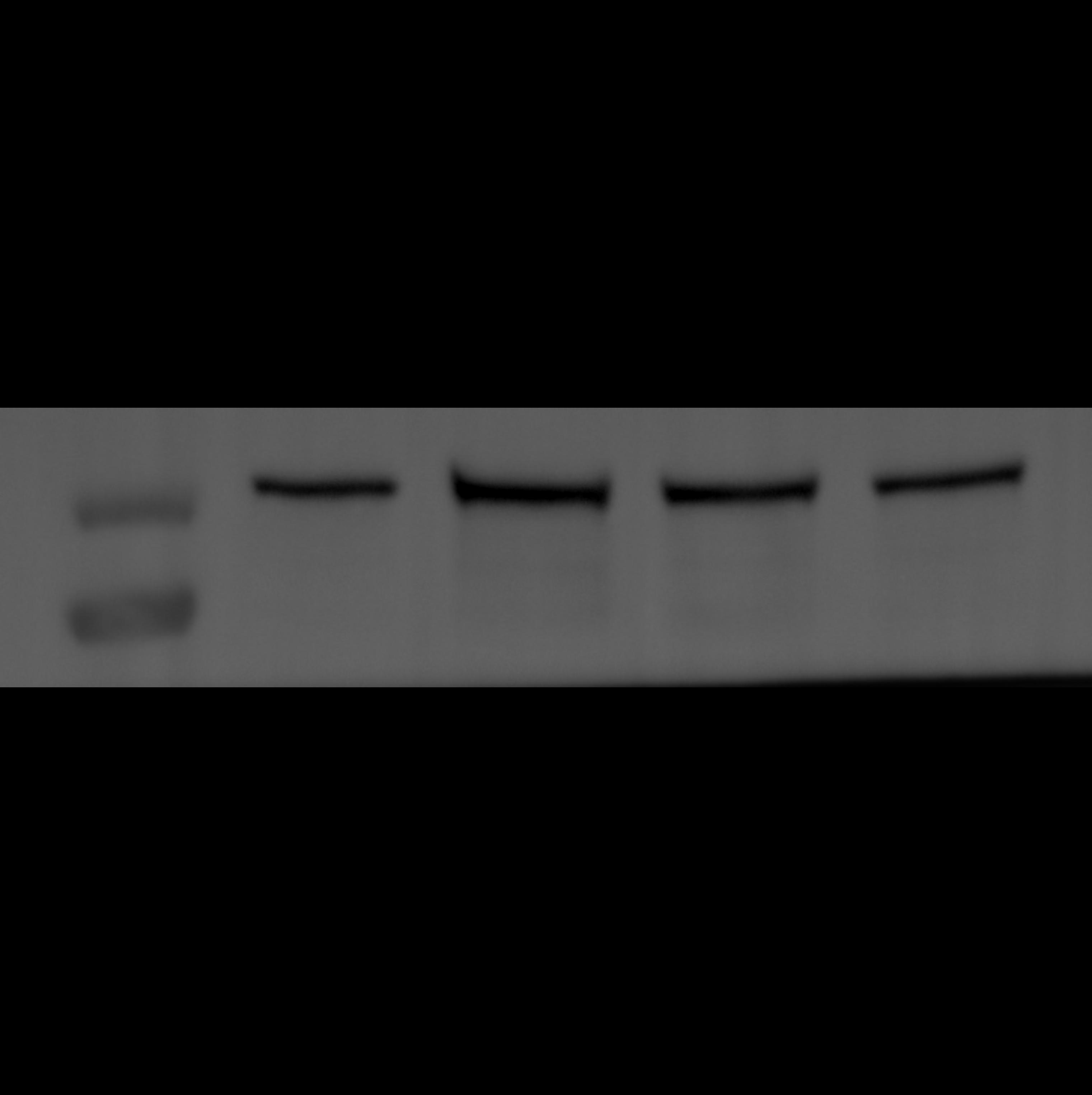

Supplement: Supplementary file 1 [file DataSheet1.zip › NLRP3/NLRP3-2-Original picture.tiff]

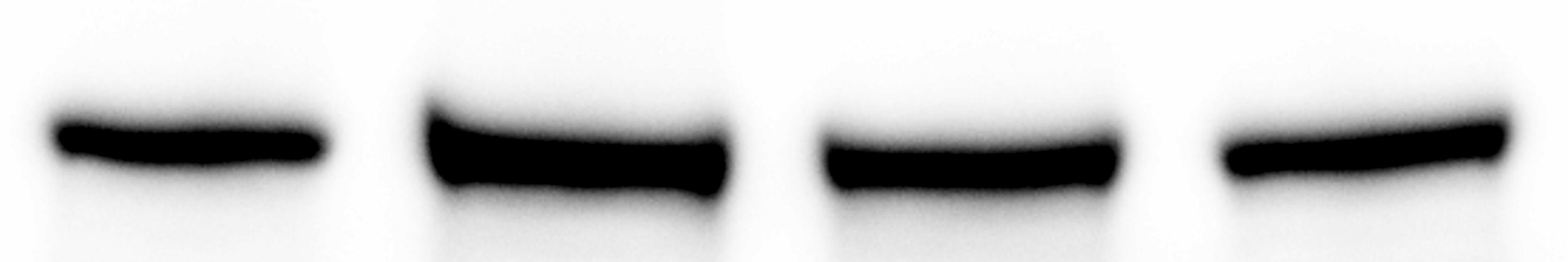

Supplement: Supplementary file 1 [file DataSheet1.zip › NLRP3/NLRP3-2.jpg]

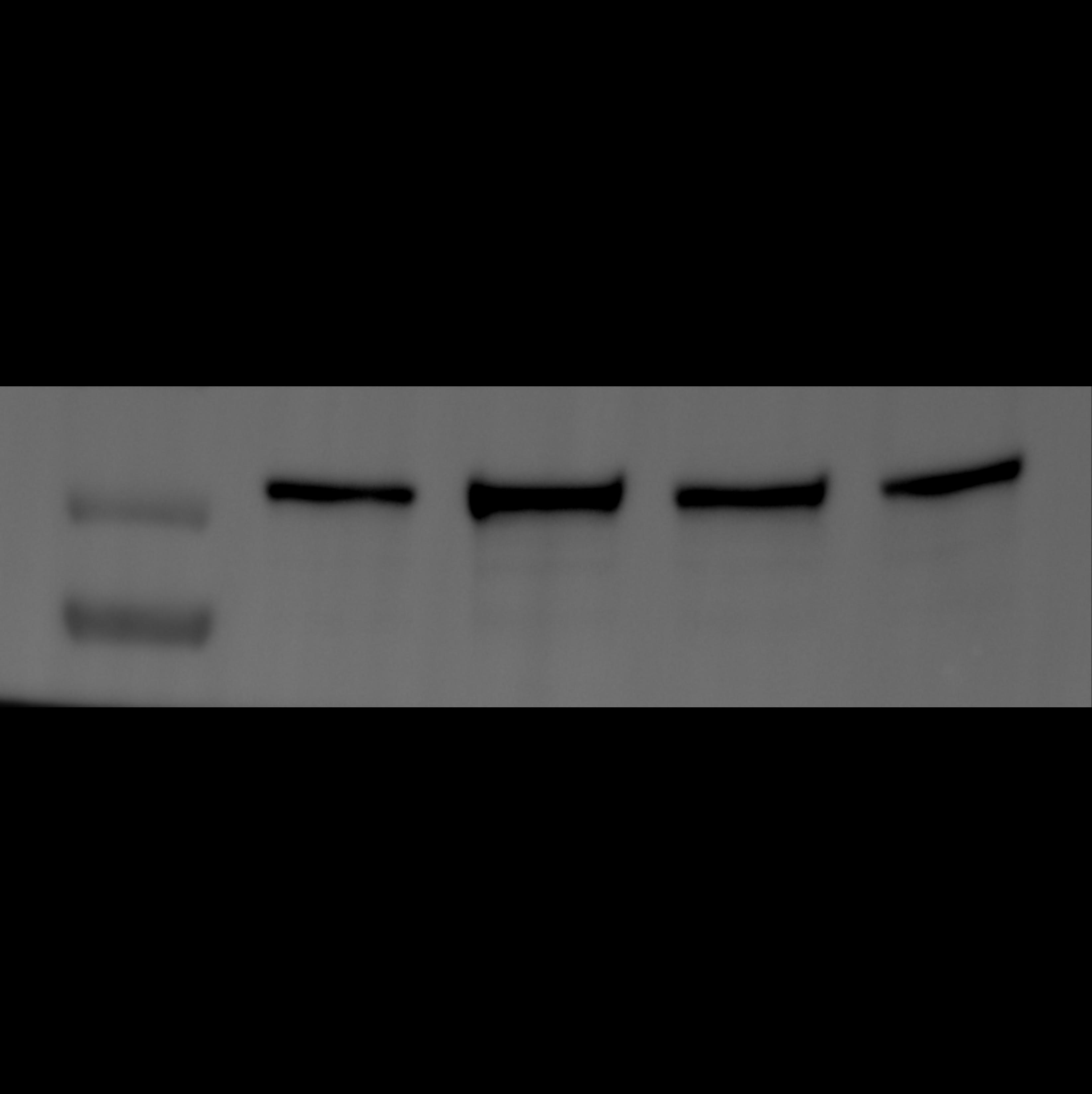

Supplement: Supplementary file 1 [file DataSheet1.zip › NLRP3/NLRP3-3-Original picture.tiff]

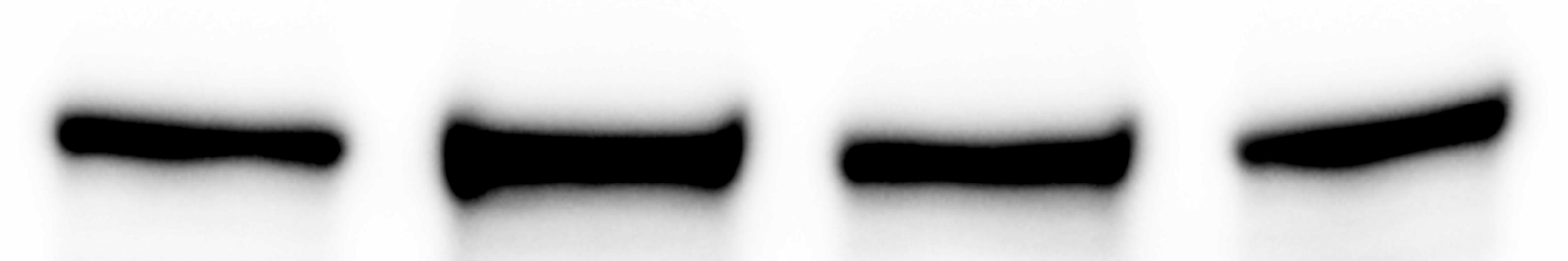

Supplement: Supplementary file 1 [file DataSheet1.zip › NLRP3/NLRP3-3.jpg]

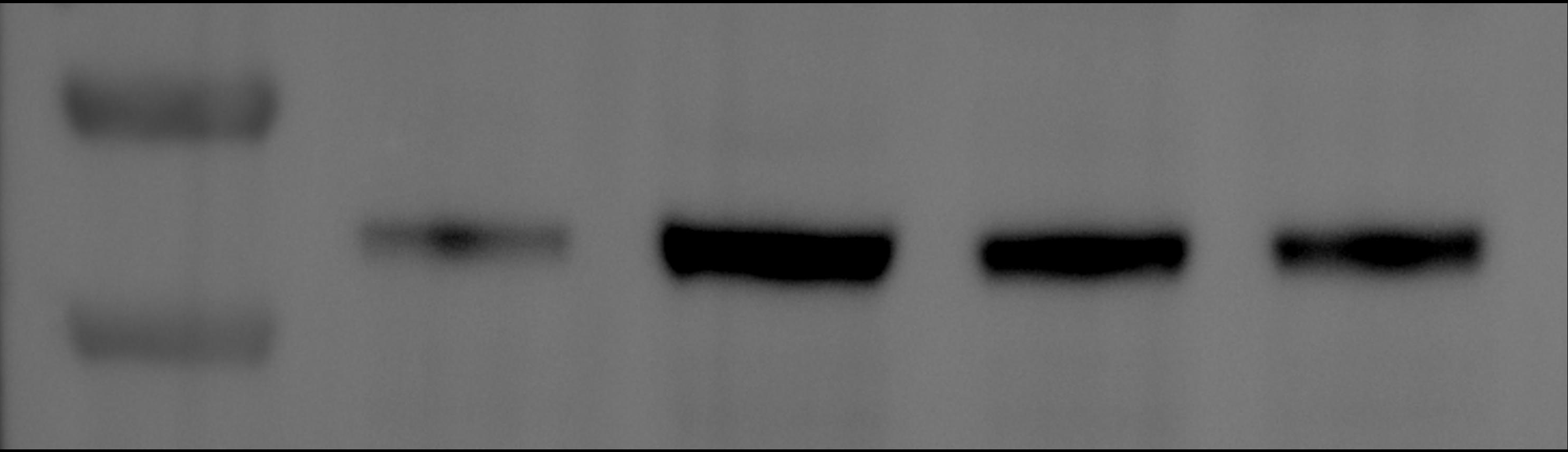

Supplement: Supplementary file 1 [file DataSheet1.zip › P62-1-Original picture.tiff]

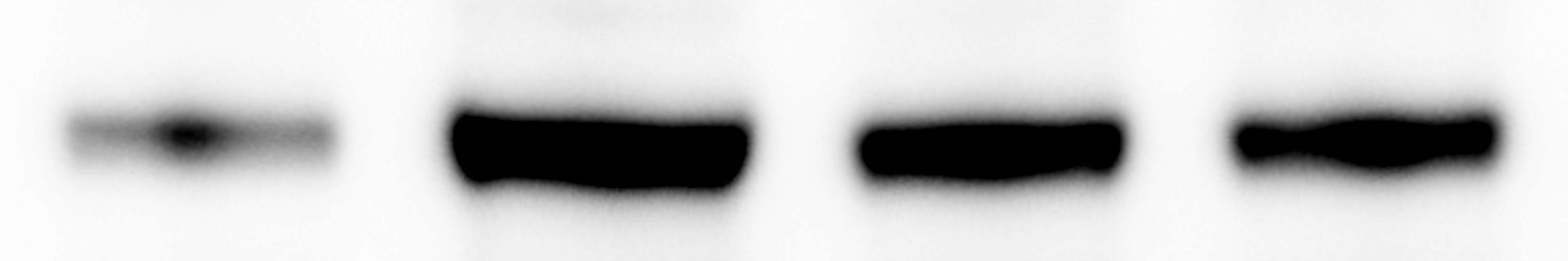

Supplement: Supplementary file 1 [file DataSheet1.zip › P62-1.jpg]

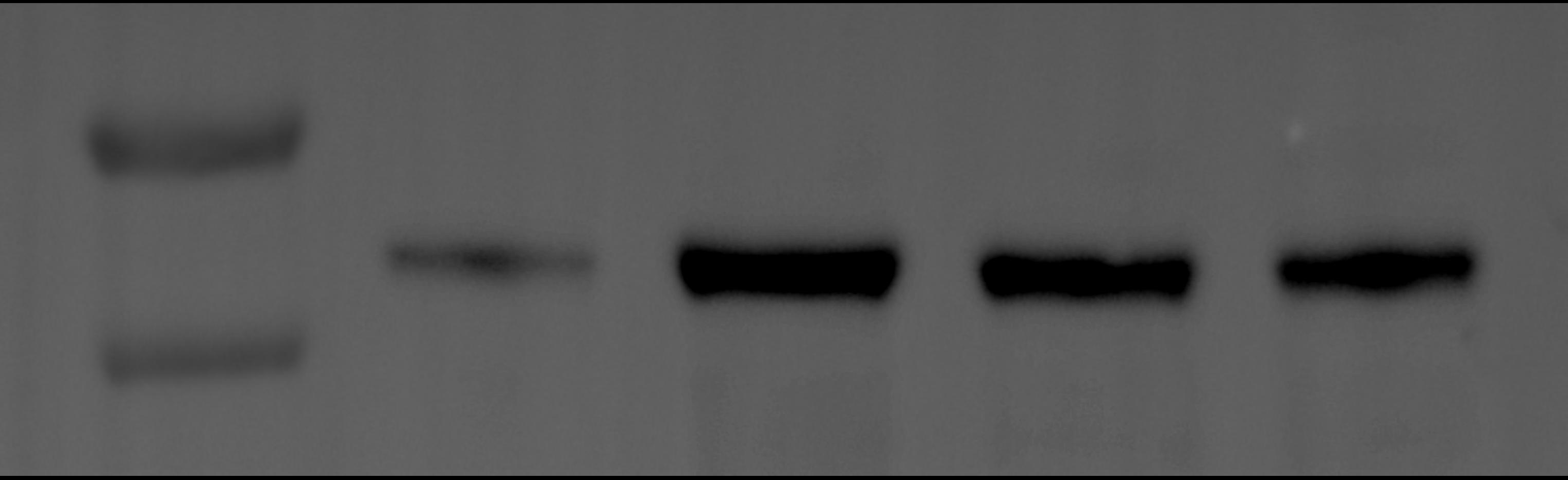

Supplement: Supplementary file 1 [file DataSheet1.zip › P62-2-Original picture.tiff]

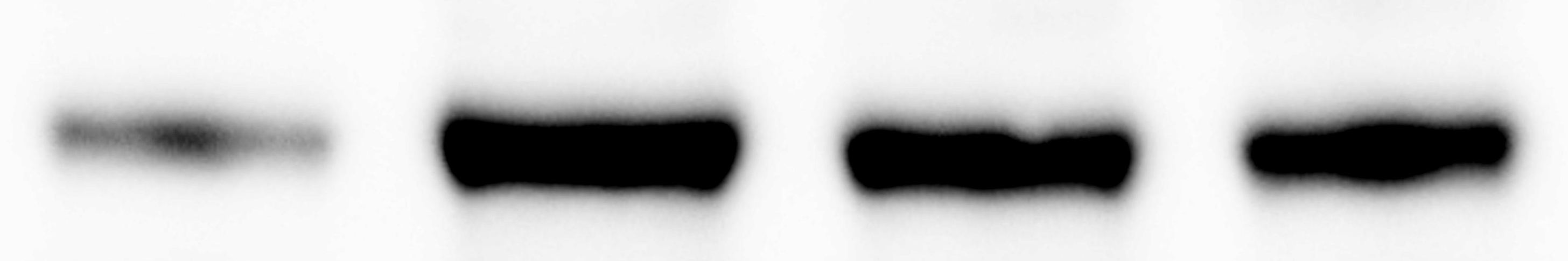

Supplement: Supplementary file 1 [file DataSheet1.zip › P62-2.jpg]

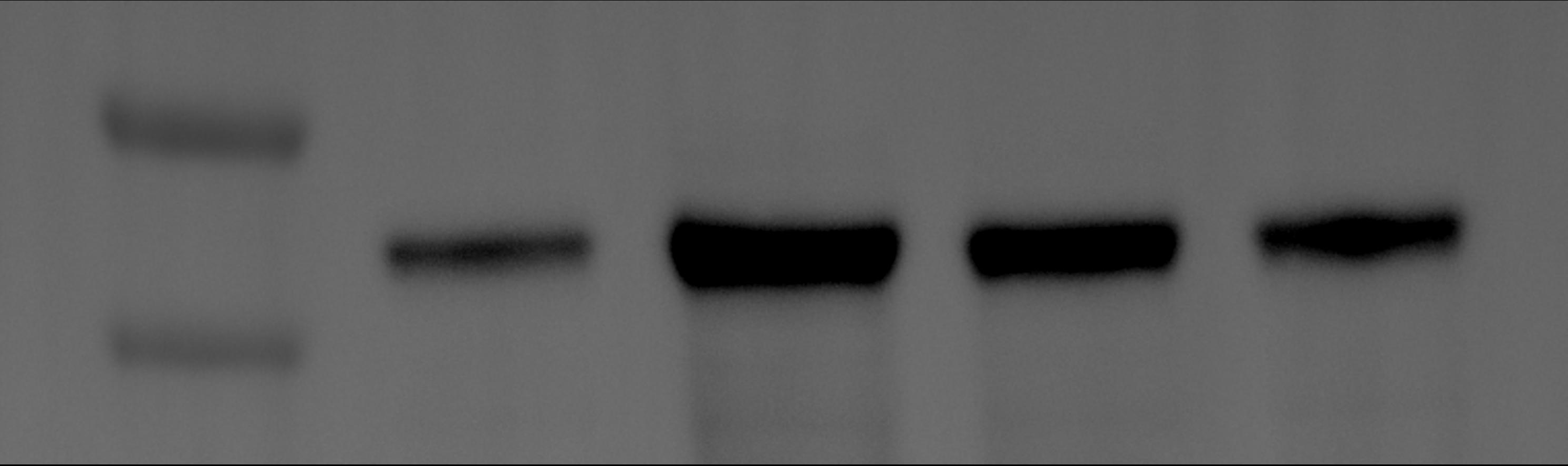

Supplement: Supplementary file 1 [file DataSheet1.zip › P62-3-Original picture.tiff]

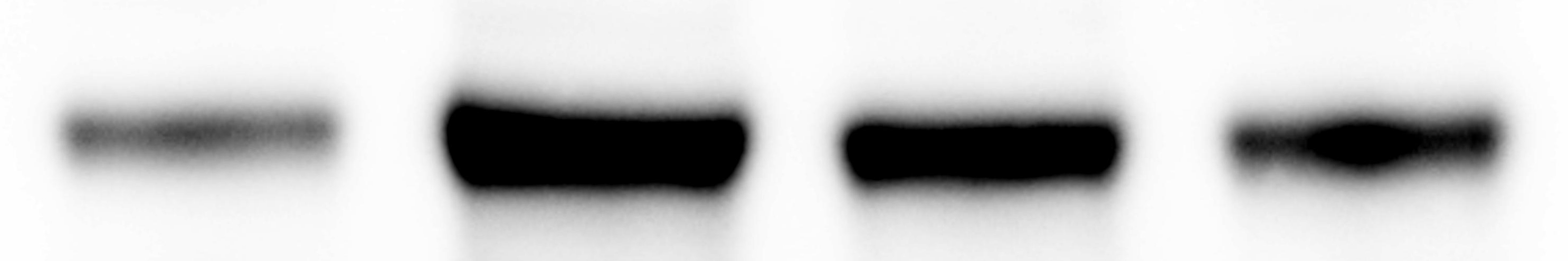

Supplement: Supplementary file 1 [file DataSheet1.zip › P62-3.jpg]
